# A spectrum of verticality across genes

**DOI:** 10.1371/journal.pgen.1009200

**Published:** 2020-11-02

**Authors:** Falk S. P. Nagies, Julia Brueckner, Fernando D. K. Tria, William F. Martin

**Affiliations:** Institute for Molecular Evolution, Heinrich Heine University Düsseldorf, Düsseldorf, Germany; National Institute of Genetics, JAPAN

## Abstract

Lateral gene transfer (LGT) has impacted prokaryotic genome evolution, yet the extent to which LGT compromises vertical evolution across individual genes and individual phyla is unknown, as are the factors that govern LGT frequency across genes. Estimating LGT frequency from tree comparisons is problematic when thousands of genomes are compared, because LGT becomes difficult to distinguish from phylogenetic artefacts. Here we report quantitative estimates for verticality across all genes and genomes, leveraging a well-known property of phylogenetic inference: phylogeny works best at the tips of trees. From terminal (tip) phylum level relationships, we calculate the verticality for 19,050,992 genes from 101,422 clusters in 5,655 prokaryotic genomes and rank them by their verticality. Among functional classes, translation, followed by nucleotide and cofactor biosynthesis, and DNA replication and repair are the most vertical. The most vertically evolving lineages are those rich in ecological specialists such as Acidithiobacilli, Chlamydiae, Chlorobi and Methanococcales. Lineages most affected by LGT are the α-, β-, γ-, and δ- classes of Proteobacteria and the Firmicutes. The 2,587 eukaryotic clusters in our sample having prokaryotic homologues fail to reject eukaryotic monophyly using the likelihood ratio test. The low verticality of α-proteobacterial and cyanobacterial genomes requires only three partners—an archaeal host, a mitochondrial symbiont, and a plastid ancestor—each with mosaic chromosomes, to directly account for the prokaryotic origin of eukaryotic genes. In terms of phylogeny, the 100 most vertically evolving prokaryotic genes are neither representative nor predictive for the remaining 97% of an average genome. In search of factors that govern LGT frequency, we find a simple but natural principle: Verticality correlates strongly with gene distribution density, LGT being least likely for intruding genes that must replace a preexisting homologue in recipient chromosomes. LGT is most likely for novel genetic material, intruding genes that encounter no competing copy.

## Author summary

Because multicellular life is a latecomer in Earth history, most of evolutionary history is microbial evolution. Scientists investigate microbial evolution by studying the evolution of genes. One of the main surprises of the genomic era is the amount of lateral gene transfer that has gone on in prokaryote genome evolution. Gene transfer clouds evolutionary history, but by how much: How lateral and how vertical is the microbial evolutionary process across genes, genomes and lineages? We introduce measures of verticality in genome evolution that permit a ranking of genes and lineages according to their degree of verticality. We show that genes already present in genomes are less likely to be replaced by a newly introduced copy than genes that offer new evolutionary opportunities for the recipient, providing a simple and natural mechanism that limits and promotes lateral gene transfer frequency. Only a very small minority of prokaryotic genes evolve vertically. While the 100 genes that are most widely used to describe the phylogenetic relationships of microbes are indeed the most vertical, they are not at all representative for the evolution of other genes. These findings have broad implications for how we understand the evolutionary process as inferred from gene trees.

## Introduction

Prokaryotes undergo recombination that is facilitated by the mechanisms of lateral gene transfer (LGT) [1,2]—transformation, conjugation, transduction, and gene transfer agents [3]. These mechanisms introduce DNA into the cell for recombination and do not obey taxonomic boundaries, species or otherwise. Over time they generate pangenomes [4,5] that are superimposed upon vertical evolution of a conserved core. About 30 genes are present in all genomes [6–9], a few more are nearly universal [10], many are found only in strains of one species [5], but the vast majority of genes are distributed between those extremes according to a power law [11]. Previous work has shown that LGT is subject to natural barriers [12,13], that LGT affects core metabolism less than it affects peripheral metabolism [14] and that LGT is affected by regulatory interaction networks [15]. LGT generates collections of genes in each genome that are of different evolutionary age [16], transferred genes are non-randomly associated [17,18], and major events of gene flux have occurred during evolution [9,19]. In principle, each gene should be transferable, because the mechanisms that introduce DNA into the cell are not selective with regard to the nature of sequences introduced, notwithstanding the CRISPR activity associated with phage defense [20]. If all genes are transferrable, what determines verticality?

At the level of strains or species, gene distributions within rapidly evolving pangenomes have been well-studied [21–25]. Less well understood are the factors that govern the distribution of genes across prokaryotic genomes at higher taxonomic levels. These reflect processes that occurred in deep evolutionary time and, in some cases, underpin the physiological identity of major prokaryotic clades. Though LGT impacts prokaryotic evolution, it does not obscure lineage identity, because despite the abundance of LGT, biologists 100 years ago were able to recognize the identity of many higher level taxa, for example Cyanobacteria and Spirochaetes [26], that we still recognize today. Hence there must exist a spectrum of verticality in prokaryote lineage evolution. It follows that a natural spectrum of verticality across prokaryotic genes should exist as well. Here we rank 101,422 gene families from 5,655 prokaryotic genomes according to conservative estimates of verticality and report how this attribute affects phylogenetic inference in microbial evolution in general and as it impacts inference of eukaryote origin in particular.

## Results

### The verticality of genes

The two main parameters influencing reconstruction of gene evolution across prokaryotes are sequence conservation and phylogenetic distribution, both of which are easy to estimate from clustering methods based on pairwise sequence comparisons. The degree of congruence among trees for overlapping leaf sets is, by contrast, determined by two unknowns: the accuracy of phylogenetic inference relative to the true gene trees, and the relative amount of LGT that has, or has not, occurred in the evolution of each gene (verticality ***V***). There are many methods of tree comparison, but not for measures of gene verticality. If a gene occurs in many lineages, one invariably observes discordance between the branching pattern generated by the gene and that generated by some standard such as rRNA, yet whether such discordance is due to LGT or to technical issues involving alignment and phylogeny [27] is virtually impossible to determine, because knowledge of the amino acid substitution process underlying sequence divergence in real alignments is irretrievable from real data [28]. That problem is exacerbated in trees having thousands of leaves, where random phylogenetic differences are inevitable. For example, there are 3 · 10^80^ possible topologies for a tree with 52 leaves, and there are about 10^80^ protons in the universe [29]. A comparison of two trees, each with 52 (or 520, or 5,200) leaves for an alignment of 400 amino acid sites, evaluates many branches that are not better than random.

Earlier surveys of lateral gene transfer across 116 prokaryotic genomes using nucleotide frequency comparisons were reported over a decade ago [30]. In the era of computers that can calculate all trees for all genes, we sought a measure of verticality that is based on phylogenetic principles but independent of the problems inherent to topological comparisons of large trees. To obtain such an estimate, we leveraged two simple but robust assumptions. First, we assume that the higher order taxa of prokaryotes (referred to here as phyla) that microbiologists have traditionally recognized based on morphological, physiological and rRNA sequence criteria are real and constitute monophyletic groups. On that premise, the null hypothesis for phylogenetic behavior of a given gene in a given prokaryotic phylum is vertical evolution (phylum monophyly). Our second assumption for estimating verticality is that molecular phylogeny works most reliably at the tips of trees, the terminal branches. This assumption is the basis of Neighbor Joining [31], almost all alignment programs [32], and maximum likelihood methods, which typically start the topology search from an NJ tree [33]. By reading the trees only at the tips, we disregard phyletic patterns in deeper branches, where pairwise sequence similarity fades and the processes underlying sequence differences, alignments, and branching pattern differences become more numerous, more poorly constrained and more prone to inference errors.

To estimate ***V***, we read the information contained in each tree solely with regard to the branching patterns of phyla by posing the following recursive set of questions: 1) For each phylum that exists in our data, do sequences from the phylum occur in the tree? 2) If so, do they form a monophyletic group (a clade) or are they singletons? 3) How many clades do they form in that tree? 4) For each clade for tree *i* and phylum *j*, what is the phylogenetic composition of the sister group? That set of questions is repeated for all phyla in tree *i*, the results are tabulated, and the procedure repeated for the next tree. The resulting data contains information both about the verticality of all genes (how often phyla appeared monophyletic for each gene) and about the verticality of genome evolution in all phyla (how often phyla were monophyletic across all genes in the phylum). In a world without LGT and perfect data that reconstructs the true tree from the alignment, all phyla would be monophyletic, all genes from the same phylum would have the same sister phylum and each gene would appear to be inherited vertically. In real data, LGT exists and the data are not perfect, but by looking only at the tips we can estimate verticality without random effects among deeper branches. Note that the true branching order of phyla relative to one another has no bearing upon our estimate of ***V***, nor does the relative branching of lower order taxa within each phylum. For a given gene, we calculate ***V*** as follows. For each tree, phyla that are not monophyletic are given a score of zero, the number of genomes present in the tree for each monophyletic phylum is divided by the number of genomes from that phylum among the 5,655 genomes in the data; that proportion is summed across all monophyletic phyla in the tree, that sum is ***V*** for that tree or cluster. For *n* phyla, ***V*** obtains a value between 0 and *n*.

This measure scores the verticality of a gene across all phyla in which it occurs and gives a higher rank to genes that recover phylum monophyly in a tree sampling many phyla than to those with a more narrow distribution, where the opportunity to observe LGT in tree tips is reduced. Note that an accurate taxonomic assignment for each gene is important for estimating ***V***, for which reason we do not include metagenomic data, where binning can lead to assemblies of genes from different lineages. Clustering all 19,050,992 genes yielded 448,821 clusters with genes spanning at least two sequenced genomes, with 261,058 clusters spanning at least three genomes for tree reconstruction with an average of 66.4 genomes and 68.7 sequences each. Removing trees that contained sequences from only one phylum left 101,422 trees containing on average 138.8 genomes and 146.7 sequences (median 18 for both).

The first question we asked was whether gene duplications are frequent, as they might emulate LGT and thus mask verticality. For smaller data sets it is known that gene duplications in prokaryotes are generally rare as compared to eukaryotes [34] and that genome sizes constrain the number of duplicates (or transfers) that a genome can accommodate [11]. Estimating ancient duplications for this data set is not possible as duplications and transfers would be indistinguishable, but recent duplications can be quantified. We found 32,277 cases in which the sister of a terminal leaf (gene) occurred within the same prokaryotic genome. For 5,655 prokaryotic genomes this yields 5.7 genome specific duplications per genome. For comparison, 150 eukaryote genomes [35] harbor 109,056 genome specific duplications corresponding to 727 genome specific duplications per genome. Thus, based upon the values for recent duplications in the present sample, gene duplications per genome are 134-fold less frequent in prokaryotes than in eukaryotes. We also plotted the fraction of terminal duplicates normalized for genome size and compared the distribution in eukaryotes versus prokaryotes using all genomes. The cumulative distribution function (**S1A Fig**) shows that a eukaryotic genome has, on average, 4% recent duplications while prokaryotes have 0.2%. This is not an effect of unequal sample size, because the average 20:1 ratio is robust for 100 random samples of 150 prokaryotic genomes (**S1B Fig**). That duplications are 20–134 fold less frequent in prokaryotes than in eukaryotes in this sample of 5,655 genomes corresponds well with the earlier estimate from six groups of closely related bacteria that ~98% of gene families in prokaryotes result from LGT, not duplication [34]. It suggests that in prokaryotic genomes, duplication (paralogy) does not impact estimates of ***V*** in prokaryotic genomes to an appreciable extent, a caveat for methods that allow and infer roughly equal probabilities of LGT and duplication, both for prokaryotes and for eukaryotes [36].

**Fig 1:**
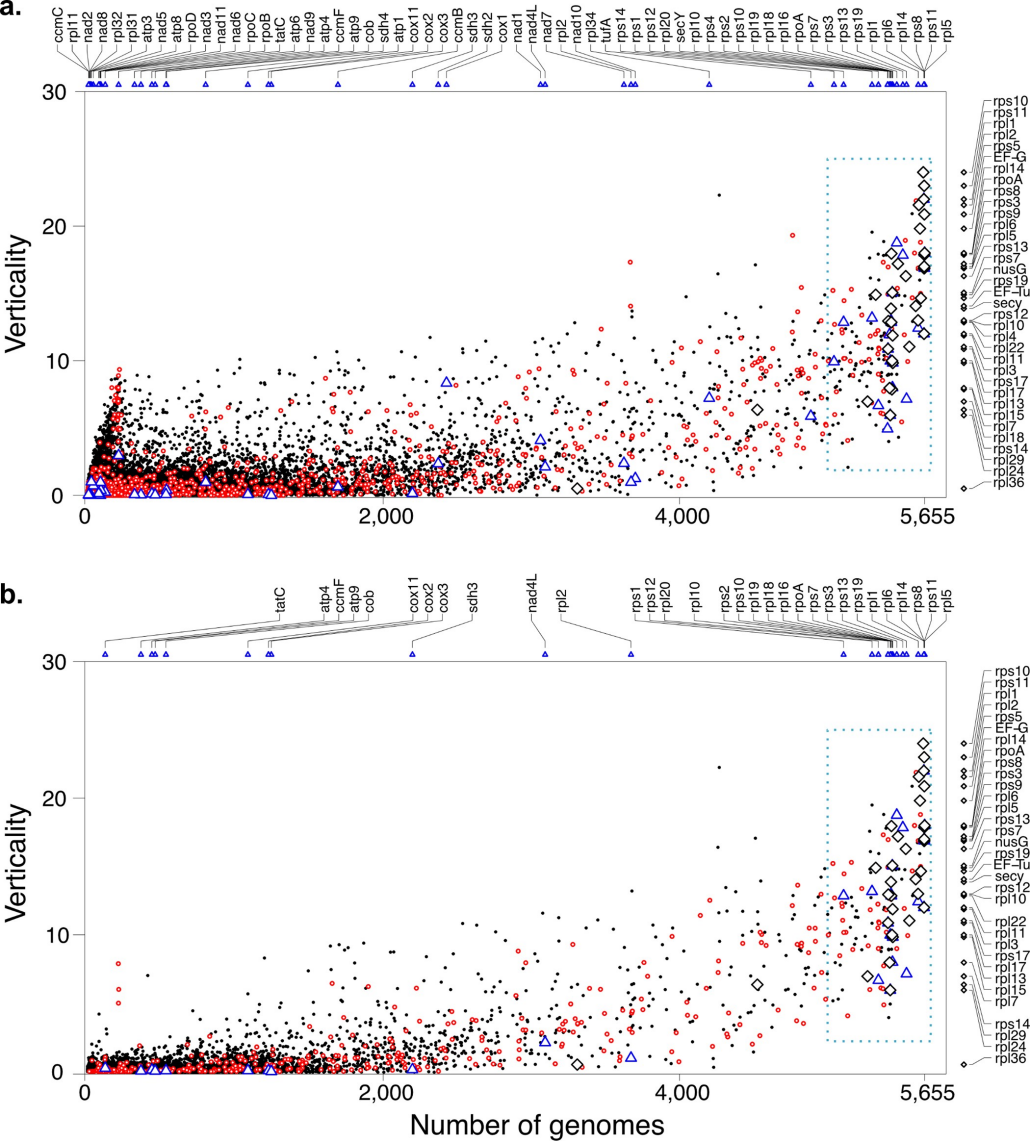
Comparison of estimated verticality and number of genomes in a protein cluster for **a.** all clusters (n = 101,422) and **b.** all conserved clusters (average branch length ≥ 0.1; n = 8,547). Unrooted trees were analyzed if at least two taxonomic groups were present. Verticality was calculated as the sum of monophyletic taxonomic groups in a cluster adjusted by the fraction of a taxonomic group represented in the cluster. The procedure for determining verticality on the basis of an example is shown in **S3 Fig**. This value correlates with the number of genomes, an approximation of universality, which is even more apparent when clusters of high evolutionary rate were filtered out (a.: p < 10^−300^, Pearson´s R^2^ = 0.726; b.: p < 10^−300^, R^2^ = 0.829). In both plots clusters of special interest were marked: The eukaryotic-prokaryotic clusters (EPCs) are highlighted in red and the clusters that correspond to a gene from the mitochondrial genome of *Reclinomonas americana* [45] are displayed in blue triangles along the abscissa of the plot and in the graph. For the latter, the gene identifier was noted above each plot. Ribosomal proteins are indicated by the black diamond on the right of each plot and in the graph [6]. Notably, the ribosomal protein clusters show a steep gradient of verticality among conserved clusters with similarly wide distribution.

The values of ***V*** obtained for all genes allows us to rank them by their relative degree of verticality or LGT, as one prefers. What governs LGT? Few factors have been suggested to govern the rate of LGT that genes undergo. It has been suggested that LGT is limited by the number of intermolecular interactions in which a molecule in involved [37]. Although many genes with high values of ***V*** encode ribosomal proteins, which have many interactions, many ribosomal proteins have modest values of ***V***. We found that the majority of highly vertical genes are soluble proteins as opposed to being components of macromolecular complexes, and that verticality ***V*** strongly correlates with the gene’s distribution frequency across genomes, as shown in **Fig 1**, where the value of ***V*** estimated for each gene is plotted against the number of genomes in which it occurs. **Fig 1A** shows the verticality and distribution of all 101,422 clusters that generate trees. **Fig 1B** displays the verticality the 8,547 clusters that contain more conserved sequences, that is, those that have an average branch length ≤ 0.1 substitutions per site. The spike of sequences at the left of **Fig 1A** represents sequences that tend to be vertically inherited within closely related lineages but whose clusters span only a few genomes because they are not well conserved, for which reason the spike, which encompasses 836 clusters (0.8%; see **S1 Table**), is not present in **Fig 1B**.

The value of ***V*** as calculated has desirable properties because it takes distribution into account. In order to see whether verticality is correlated with distribution, we also calculated values of verticality that are independent of distribution, using the number of monophyletic phyla per tree multiplied by the average root-to-tip distance [38] (weighted verticality, ***V***_***w***_; **S10 Table**) instead of dividing by the number of phyla in which the gene is present. The correlation between gene distribution frequency and weighted verticality ***V***_***w***_ as inferred independent of distribution frequency was significant at *p* < 10^−300^ (**S2 Fig**, **S2 Table**). From that one obtains a very general observation about verticality and gene distribution: The most densely distributed genes tend to have the highest verticality, that is, the lowest frequency of recent LGT as determined by phylogenetic criteria.

Why should the most densely distributed genes tend to be most resistant to LGT? We suggest that the reason is simple: If a well-regulated, codon-bias adapted [2] resident copy of a gene already exists in the genome, it would have to be displaced by the intruding copy. Selection in prokaryotes can be intense, as evidenced by codon bias itself: synonymous substitutions that impair codon bias for highly expressed genes are tenaciously counter selected in nature [2]. The existence of a preexisting copy of a gene in the genome reduces the probability of LGT in a highly significant manner (R^2^ = 0.726; **Fig 1B**). This is all the more noteworthy because the genes that most frequently enter a recipient cell via LGT in nature will be those that are themselves the most widespread genes in nature—that is, the most common genes will be introduced into recipients with the highest frequency. Prokaryotic genes thus have a home field advantage relative to intruders.

The mechanisms of LGT (transduction, transformation, conjugation, gene transfer agents) operate constantly across all prokaryotic genomes in the wild. All things being equal, new coding sequences enter the prokaryotic genome as a random sample of genes available in the environment [39,24], producing natural variation in gene content upon which selection and drift [40] can act to prolong or curtail the gene’s lifespan, or residence time, in the descendant clonal lineage. Genes that interfere with the workings of the cell [13] are eliminated quickly from the accessory genome and therefore have a short residence time. Neutral genes that merely constitute functionless ballast can persist in the pangenome longer before loss, while genes that are of value under circumstances encountered by the recipient can become fixed [23,24], in which case they start to shift from the accessory genome to the core genome, thereby defining new genomic lineages of vertical core descent.

The gene families that we observe to be the most vertical (**Fig 1**, **S1 Fig**) are those that are most widely distributed among genomes and hence the most prevalent in nature. This would be puzzling were it not for an inhibitory effect that presence of a preexisting copy exerts on the success rate of LGT. Transposases constitute a special case. They are likely the most common genes in nature [41], but there are different classes of transposases [41], hence they do not fall into one cluster. The fate of transposases is not governed by selection and drift, as they self-amplify within genomes, increasing their copy number by virtue of their ability to do so [42], not by virtue of selection and drift.

**Fig 2:**
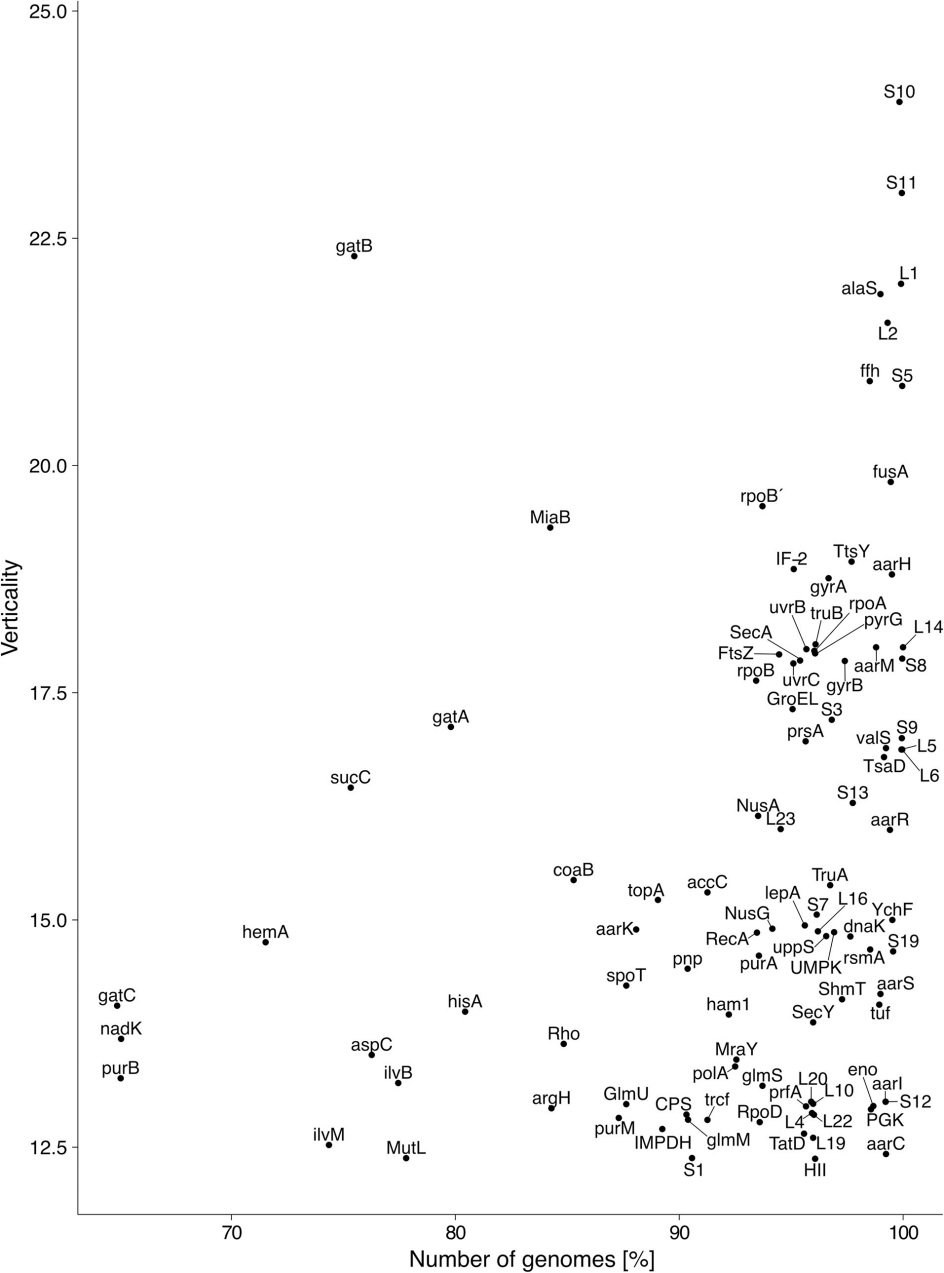
Comparison of estimated verticality and number of genomes [%] for the 100 most vertical clusters. Identity and Annotation of clusters can be found in **S6 Table**. This is a representation of some of the clusters shown in the blue rectangle of **Fig 1A**.

The verticality of genes has practical importance for prokaryotic phylogeny, because modern approaches to prokaryotic systematics typically aim to increase the amount of information per lineage beyond that provided by ribosomal RNA. Since 1997, phylogenetic studies of prokaryotic genomes have typically concatenated dozens of sequences into longer alignments [6,43,44]. However, it is not enough to just combine sequences into longer alignments, the sequences ideally need to share the same evolutionary history. ***V*** provides a measure for how vertically a gene tends to evolve over evolutionary time spans. Ranking all genes by their verticality (**Fig 1**; **S1 Table**) provides criteria for inclusion of genes for phylogenetic studies. For orientation, in **Fig 1** we have labelled along the ordinate the genes in current use for phylogenetic studies of archaeal lineages and their relationship to the host that acquired the mitochondrion at eukaryote origin [45]. They differ in their degree of verticality. A number of sequences that are not widely used for phylogeny exhibit higher verticality; these are shown in **Fig 2** and listed in **S6 Table**. Similarly, genes encoded in mitochondrial DNA are typically used to investigate the relationship of mitochondria to bacterial lineages [46]. Those genes are a subset of the genes found in *Reclinomonas americana* mitochondrial DNA [47], which are indicated along the abscissa in **Fig 1**.

From the standpoint of phylogenetics, the main message of **Fig 1** is twofold. First, the genes most commonly used as markers in broad scale prokaryotic phylogenetic studies are, in terms of their distribution and their verticality, not representative for the genome as a whole. Worse, without the comparative information from **Fig 1** they could even be positively misleading, because without measures to compare verticality across genes, one might assume that the tendency of the most widely distributed genes to be vertically inherited is representative for the phylogenetic behavior of all genes. But that is not the case. Widely distributed genes tend to be vertically inherited but they are not a representative sample for the phylogenetic behavior of the genome as a whole. The vast majority of prokaryotic genes are not inherited vertically, hence the small vertically inherited sample is not a good proxy for the phylogenetic behavior of prokaryotic genes. Vertically inherited genes in prokaryotes are not a random sample, they are a biased sample. This is also known as the tree of 1% [9] and is most clearly seen in **Fig 1B**, where the more conservatively evolving, hence phylogenetically more useful genes are shown. The vast majority of genes that occur in two or more phyla in prokaryotes fail to recover phylum monophyly to any appreciable extent, also for estimates of ***V*** that are independent of distribution (**S2 Fig**), and most of them are present in only very few phyla to begin with. The mean and median values of ***V*** in **Fig 1A** are 0.27 and 0.04, in **Fig 1B** 0.70 and 0.06, respectively. The second main message of **Fig 1** concerns the relationship of eukaryotic clusters to prokaryotic clusters. We mapped these prokaryotic clusters to eukaryotic clusters (see **Methods**) as indicated by red circles in **Fig 1**. Their significance will be discussed in a later section.

### The most vertical and lateral genes and categories

**Table 1** lists the 20 most vertically and 20 least vertically inherited genes in sequenced prokaryotic genomes, both for the complete sample and for the conserved fraction of genes. Among the most vertical are the ribosomal proteins, ribosomal protein S10 currently being the most vertical protein in genomes, followed by other proteins involved in information processing. The least vertically inherited genes by our conservative tip-based approach, comprise various categories (**Table 1**), the complete lists of genes ranked by verticality is given in **S1 Table**.

**Table 1:** Maximum likelihood trees from 19,050,992 protein sequences from 5,433 bacterial and 212 archaeal species were calculated for clusters obtained by MCL, yielding 101,422 trees with at least four sequences and two taxonomic groups present. Each of the 101,422 trees were assigned a protein label according to the NCBI sequence header that was represented the most. On the left panel all trees were annotated and sorted according to their verticality score for the genes (*V*_*g*_). The number of organisms in the respective cluster is stated as *N*_spec_. On the right panel the same values are stated only for conserved protein families–determined by average branch length ≤ 0.1.

	All 101,422 protein families			The 8,547 most conserved protein families		
	*V*_*g*_	Protein family	*N*_spec_	*V*	Protein family	*N*_spec_
**Most vertical**						
	24.00	30S ribosomal protein S10	5,646	24.00	30S ribosomal protein S10	5,646
	23.00	30S ribosomal protein S11	5,652	23.00	30S ribosomal protein S11	5,652
	22.30	Asp/glu–tRNA amidotransferase subunit B	4,269	22.30	Asp/glu–tRNA amidotransferase subunit B	4,269
	22.00	50S ribosomal protein L1	5,650	22.00	50S ribosomal protein L1	5,650
	21.89	Alanine–tRNA ligase	5,598	21.89	Alanine–tRNA ligase	5,598
	21.57	50S ribosomal protein L2	5,616	21.57	50S ribosomal protein L2	5,616
	20.93	Sec family type I SRP^a^ protein	5,571	20.93	Sec family type I SRP^a^ protein	5,571
	20.88	30S ribosomal protein S5	5,653	20.88	30S ribosomal protein S5	5,653
	19.82	Translation elongation factor G	5,624	19.82	Translation elongation factor G	5,624
	19.55	DNA-directed RNA polymerase subunit beta	5,300	19.55	DNA-directed RNA polymerase subunit beta	5,300
	19.32	tRNA methylthiotransferase MiaB	4,764	18.86	Translation initiation factor IF-2	5,379
	18.94	Signal recognition particle-docking protein FtsY	5,525	18.80	Histidine–tRNA ligase	5,627
	18.86	Translation initiation factor IF-2	5,379	18.76	DNA gyrase subunit A	5,467
	18.80	Histidine–tRNA ligase	5,627	18.00	50S ribosomal protein L14	5,655
	18.76	DNA gyrase subunit A	5,467	18.00	Methionine–tRNA ligase	5,587
	18.03	tRNA pseudouridine synthase B	5,434	17.98	Excinuclease ABC subunit B	5,411
	18.00	50S ribosomal protein L14	5,655	17.96	DNA-directed RNA polymerase subunit alpha	5,431
	18.00	Methionine–tRNA ligase	5,587	17.93	CTP synthetase	5,433
	17.98	Excinuclease ABC subunit B	5,411	17.88	30S ribosomal protein S8	5,653
	17.96	DNA-directed RNA polymerase subunit alpha	5,431	17.85	Preprotein translocase subunit SecA	5,395
**Most lateral**						
	0	Heavy metal-responsive transcriptional regulator	2,392	0	SDH cyt b556 large subunit	2,344
	0	SDH cyt b556 large subunit	2,344	0	RnfH family protein	2,004
	0	Anaerobic ribo.-triP^b^ reductase activating protein	2,078	0	Hypothetical protein	1,964
	0	Thiol:disulfide interchange protein DsbC	1,952	0	Amino acid ABC transporter permease	1,666
	0	RnfH family protein	2,004	0	Succinate dehydrogenase, HM^c^ anchor protein	1,800
	0	Disulfide bond formation protein B 1	1,808	0	LysR family transcriptional regulator	1,267
	0	Hypothetical protein	1,964	0	Hypothetical protein	1,688
	0	Amino acid ABC transporter permease	1,666	0	Maleylacetoacetate isomerase	1,430
	0	LysR family transcriptional regulator	1,431	0	Sigma-E factor regulatory protein RseB	1,599
	0	Succinate dehydrogenase, HM^c^ anchor protein	1,800	0	tRNA synthase TrmP	1,567
	0	LysR family transcriptional regulator	1,267	0	tRNA 5-methoxyuridine(34) synthase CmoB	1,525
	0	Hypothetical protein	1,688	0	Chemotaxis phosphatase CheZ family protein	1,483
	0	Maleylacetoacetate isomerase	1,430	0	Hypothetical protein	1,505
	0	Sigma-E factor regulatory protein RseB	1,599	0	Hypothetical protein	1,345
	0	tRNA synthase TrmP	1,567	0	Outer membrane protein assembly protein	1,301
	0	tRNA 5-methoxyuridine(34) synthase CmoB	1,525	0	Deoxyribonuclease I	1,269
	0	Chemotaxis phosphatase CheZ family protein	1,483	0	Formate dehydrogenase accessory protein FdhE	1,241
	0	Hypothetical protein	1,505	0	Flagellar export protein FliJ	1,208
	0	Hypothetical protein	1,345	0	Hypothetical protein	1,200
	0	Hypothetical protein	1,325	0	Hypothetical protein	1,179

Notes

^a^ SRP protein–general secretory pathway protein signal recognition particle protein

^b^ ribo.-triP–ribonucleoside-triphosphate

^c^ HM–hydrophobic membrane

Although we have no estimate of ***V*** for rRNA, as its sequence in part defines phyla, the tendency we see for widely distributed protein coding genes to resist LGT would also explain why rRNA is itself so refractory to transfer [13,48], the rRNA genes that are present in a recipient genome are difficult to improve upon or match in functional efficiency, and the rRNA gene product can comprise up to 20% of the cell’s dry weight [49]. Genes for rRNA thereby carry great inertia against LGT and are therefore difficult to displace by intruding copies. The rank of functional categories (**Table 2**) with respect to verticality reveals that the clusters functionally associated with translation rank highest, followed by nucleotide metabolism (many proteins without intermolecular interactions), replication, folding and vitamin synthesis. Genes for vitamin synthesis are not highly expressed but are widely distributed and are highly vertical. The least vertical categories comprise drug resistance and community interactions. Cognoscenti might surmise that there are no real surprises in the ranking of functional categories with respect to ***V***, an indication that our measure of ***V*** is recovering meaningful information about gene evolution.

**Table 2:** Assignment of KEGG level B functional annotations. On the left panel all prokaryotic maximum likelihood trees were annotated and sorted according to their average verticality score (*V*_avg_). The number of clusters employed for this analysis are indicated (*N*_clust_). The same procedure was performed on the right panel only for conserved protein families–determined by average branch length ≤ 0.1.

All 101,422 protein families			The 8,547 most conserved protein families		
Function	*V*_avg_	*N*_clust_	Function	*V*_avg_	*N*_clust_
Translation	5.31	2,428	Translation	14.82	284
Metabolism of cofactors and vitamins	4.86	2,443	Nucleotide metabolism	10.21	160
Nucleotide metabolism	4.28	1,419	Metabolism of cofactors and vitamins	7.95	199
Amino acid metabolism	3.83	3,777	Carbohydrate metabolism	7.23	534
Carbohydrate metabolism	3.63	4,836	Replication and repair	7.11	187
Biosynthesis of other secondary metabolites	3.62	507	Energy metabolism	7.07	208
Glycan biosynthesis and metabolism	3.42	3,349	Amino acid metabolism	7.06	438
Metabolism	3.31	4,260	Folding, sorting and degradation	6.77	118
Energy metabolism	3.28	2,705	Metabolism of other amino acids	5.87	81
Xenobiotics biodegradation and metabolism	3.26	1,606	Metabolism	5.67	337
Replication and repair	3.14	3,502	Enzyme families	5.53	164
Transport and catabolism	3.02	2,843	Biosynthesis of other secondary metabolites	5.50	25
Metabolism of terpenoids and polyketides	2.97	1,473	Xenobiotics biodegradation and metabolism	5.36	103
Metabolism of other amino acids	2.95	745	Glycan biosynthesis and metabolism	5.33	158
Transcription	2.84	7,245	Signal transduction	5.10	240
Folding, sorting and degradation	2.79	1,873	Membrane transport	4.69	1,431
Lipid metabolism	2.65	2,864	Cell motility	4.37	124
Enzyme families	2.59	3,735	Metabolism of terpenoids and polyketides	4.31	85
Cellular processes and signaling	2.49	3,905	Transport and catabolism	4.31	143
Signal transduction	2.48	6,712	Lipid metabolism	4.20	215
Membrane transport	2.46	19,992	Transcription	4.12	409
Genetic information processing	2.31	4,838	Cellular processes and signaling	3.75	257
Cellular community prokaryotes	2.21	3,986	Cellular community prokaryotes	3.55	172
Drug resistance	2.15	1,754	Genetic information processing	3.23	269
Cell motility	1.94	3,620	Drug resistance	3.10	88
Poorly characterized	1.41	178,665	Poorly characterized	1.68	2,970

### The verticality of phyla

By averaging the verticality of all genes that occur in a given phylum, we can also estimate the verticality of phyla and rank them accordingly. This is shown in **Table 3**, for bacteria and archaea separately, where P_mono_ indicates the proportion of trees in which the given phylum was monophyletic. No phyla were always monophyletic, with values of P_mono_ topping out at about 0.8, meaning that the phylum was monophyletic in 80% of the trees in which its sequences occurred. At the level of phyla, for all genes and for the conserved genes, Acidithiobacilli emerge as the most vertically evolving bacteria, while the Thermococcales and Methanococcales emerge as the most vertically evolving archaea. The most laterally evolving bacteria are the Erysipelotrichia, a group of firmicutes related to Clostridia, and the Clostridia themselves for all genes, while for the conserved genes, the Gammaproteobacteria finish last when it comes to avoiding LGT. The archaea most riddled by LGT are the halophiles, which are methanogens that acquired their respiratory chain and aerobic lifestyle from bacteria [19]. Though not strict, there is a clear tendency for bacteria with a specialist lifestyle to resist LGT, and a tendency for generalists like the divisions of the proteobacteria to harbor less vertically evolving chromosomes, that, is to undergo LGT.

**Table 3:** Verticality of prokaryotic taxa across protein families with at least two taxonomic groups. The list of bacterial (top) and archaeal (bottom) taxa occurring in all trees (right) and only trees that were filtered for conservation (average branch length in the tree ≤ 0.1) (left). Archaeal and bacterial phyla with less than 5 representative species in the dataset were excluded. P_mono_ refers the proportion of monophyletic trees. *N*_mono_ indicates the number of trees in which this taxon is monophyletic whereas *N*_trees_ shows the number of occurrences of the phyla in the respective dataset.

		All trees– 101,423				Conserved trees– 8,547		
	Taxon	P_mono_	*N*_mono_	*N*_trees_		P_mono_	*N*_mono_	*N*_trees_
**Bacteria**								
	Acidithiobacillia	0.81	1,677	2,067		0.91	629	688
	Chlamydiae	0.74	1,378	1,867		0.75	482	642
	Tenericutes	0.68	2,770	4,076		0.50	391	776
	Actinobacteria	0.60	30,050	49,958		0.37	1,214	3,293
	Bacilli	0.59	24,365	41,526		0.25	1,017	3,997
	Chlorobi	0.59	1,728	2,946		0.80	494	619
	Thermotogae	0.57	2,252	3,937		0.65	495	764
	Cyanobacteria	0.56	8,655	15,446		0.64	843	1,319
	Deinococcus-Thermus	0.54	3,156	5,858		0.63	705	1,113
	Synergistetes	0.53	1,001	1,872		0.70	484	692
	Epsilonproteobacteria	0.52	3,815	7,270		0.37	513	1,397
	Fusobacteria	0.51	1,805	3,516		0.60	717	1,194
	Spirochaetes	0.50	5,063	10,130		0.44	683	1,564
	Bacteroidetes	0.49	11,677	23,755		0.40	759	1,879
	Gammaproteobacteria	0.48	29,439	61,803		0.18	1,078	5,874
	Negativicutes	0.45	1,892	4,170		0.59	804	1,371
	Nitrospirae	0.43	1,377	3,180		0.47	359	762
	Alphaproteobacteria	0.43	18,086	41,953		0.35	1,312	3,735
	Aquificae	0.43	1,210	2,826		0.43	290	672
	Planctomycetes	0.40	1,755	4,399		0.55	533	961
	Chloroflexi	0.39	2,349	6,003		0.46	521	1,141
	Acidobacteria	0.38	1,789	4,666		0.58	625	1,077
	Betaproteobacteria	0.38	14,203	37,225		0.34	1,601	4,775
	Deltaproteobacteria	0.37	8,512	23,013		0.38	1,005	2,618
	Verrucomicrobia	0.36	1,146	3,152		0.56	601	1,067
	Clostridia	0.32	7,481	23,638		0.34	1,084	3,196
	Erysipelotrichia	0.17	344	2,001		0.43	451	1,058
**Archaea**								
	Thermococcales	0.73	2,482	3,380		0.79	271	341
	Methanococcales	0.73	1,612	2,220		0.83	236	283
	Methanobacteriales	0.68	1,949	2,857		0.79	282	356
	Sulfolobales	0.66	2,223	3,387		0.75	280	374
	Archaeoglobales	0.62	1,415	2,286		0.79	252	318
	Methanomicrobiales	0.60	1,616	2,693		0.74	301	406
	Methanosarcinales	0.60	3,392	5,654		0.63	318	503
	Thermoproteales	0.55	1,537	2,775		0.61	257	420
	Thermoplasmatales	0.49	662	1,364		0.58	212	366
	Desulfurococcales	0.41	852	2,072		0.44	130	298
	Natrialbales	0.32	1,459	4,503		0.42	246	588
	Haloferacales	0.27	980	3,593		0.40	205	513
	Halobacteriales	0.20	1,024	5,057		0.30	178	591

**Fig 3:**
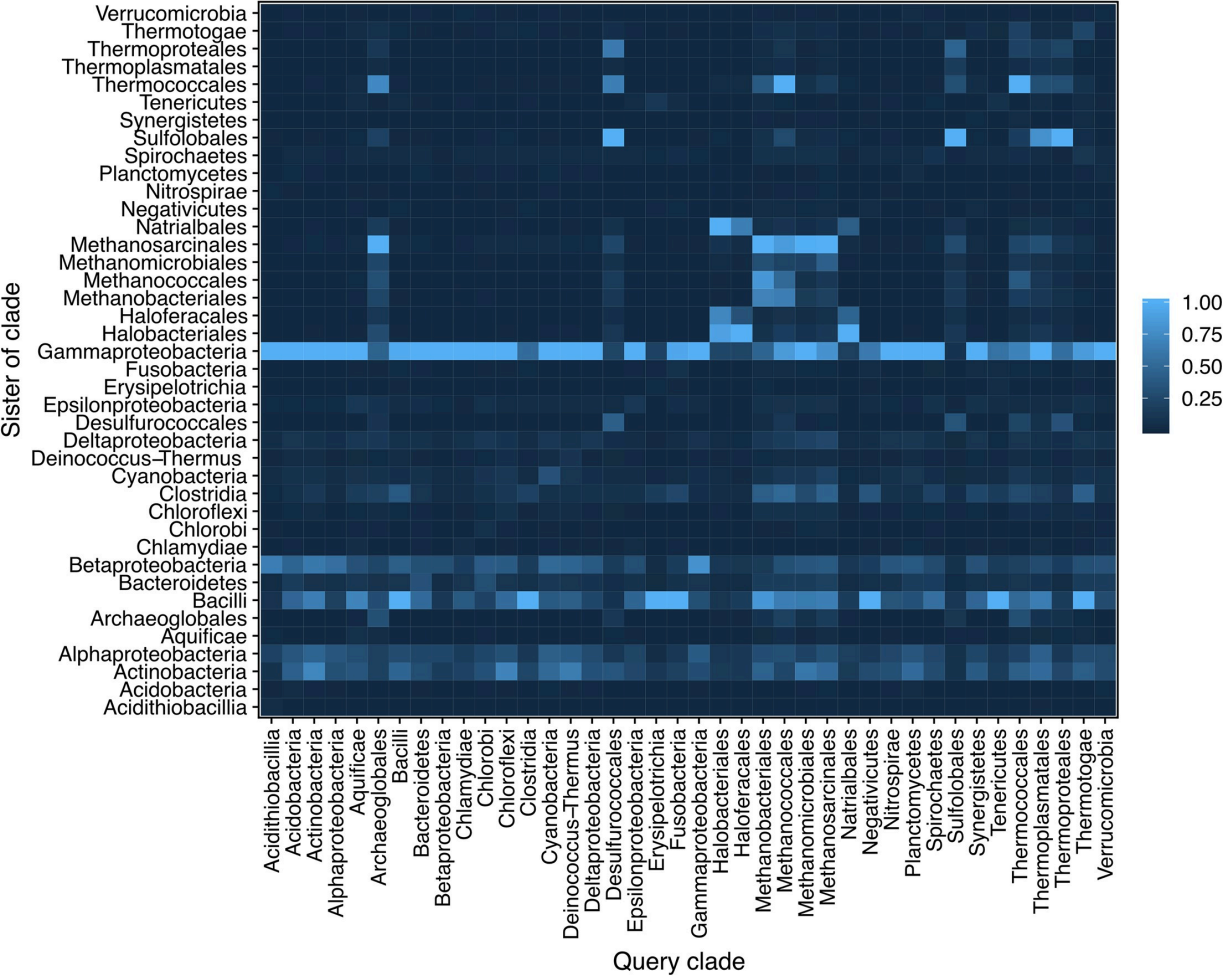
Relative occurrence of a taxonomic group as the sister group of each clade in the unrooted trees. For each taxonomic group in a cluster the sister was determined and counted. Multiple occurrences of different groups in the sister were accounted for by their relative occurrence. If the taxonomic group was paraphyletic, each monophyletic subgroup was determined and the sister of these were noted. The values of these subgroups were added up by multiplying the individual values of the sister by the fraction of the subgroup of the whole taxonomic group. To compare, the final values of each taxonomic group were normalized by dividing by the highest count a possible sister has gotten. It is apparent that Gammaproteobacteria are always overrepresented. It is not clear if the observed effects are due to overrepresentation of certain taxa in the data set or due to relative abundance of LGT.

The Gammaproteobacteria were the worst offenders when it came to LGT among the 8,547 conserved gene trees, showing gammaproteobacterial monophyly in less than 20% of trees that contained the phylum. Of course, it is possible that verticality is violated by recurrent exchanges among specific pairs of taxa or by phylogenetic artefact involving true neighbors, which for Gammaproteobacteria would be the Betaproteobacteria in traditional schemes. In order to check for such effects, each time we scored a tip-resident clade in our trees, we also scored the phylogenetic membership within its sister group. A sister group can either itself be monophyletic, containing sequences from only one phylum, or it can be mixed, containing sequences from two or more different phyla. The summary is shown in **Fig 3**, where the frequencies of phyla in the sister group are shown. Note that a phylum can appear as its own sister group when its monophyletic clade is broken by recent LGT to a member of a different phylum: the gene tree does not change, but the taxon label of the donated gene does, leaving sequences of the donor phylum that branch below the recent export in the sister group. This is illustrated in **S3d Fig**. While methanogens and halophilic archaea tend to interleave, as do archaea as a whole, the dominant signal in the sister group plot is that Gammaproteobacteria tend to be the sister of virtually every phylum, meaning that they are the recipient of genes from all phyla in our sample. The tendency to undergo recent LGT—recent because we are only scoring terminal branches—is also clearly manifest in Bacilli, Betaproteobacteria, Alphaproteobacteria and Actinobacteria, all of which harbor lineages with large genomes, large pangenomes, and diverse generalist lifestyles.

### The verticality of individual genomes

Averaging the value of verticality across all genes in a genome gives an estimate for the verticality of the genome, ***V***_g_. The verticality of all genomes investigated here is given in **S4 Table**. The most vertical genomes are those with the highest proportion of genes involved in translation. This is because the process of reductive genome evolution [50] always hones in on the ribosome, translation and information processing, as these functions are prerequisite to gene expression. The widely distributed genes involved in information processing are the most vertical (**Table 1**), such that the gammaproteobacterial endosymbiont *Carsonella ruddii* [51] which possesses only 166 protein coding genes, is the most vertical genome in our sample with ***V***_g_ = 9.44. Conversely, the least vertical genomes are the largest, with the actinobacterium *Amycolatopsis mediterranei* (***V***_g_ = 0.84) having a genome over 10 Mb coming in last. Among the archaea, the most vertical genomes were those of H_2_ dependent autotrophs (**S4 Table**). The most vertical genome was that of the highly reduced free living archaeon, *Ignicoccus hospitalis* [52] (***V***_g_ = 4.10) an extreme specialist that grows only on H_2_ + S^0^, followed by nine H_2_ dependent methanogens, starting with the thermophilic methanogen *Methanothermus fervidus* (***V***_g_ = 4.09), with a genome of 1.2 Mb [53]. The most lateral archaeal genome was that of the halophile *Haloterrigena turkmenica* (***V***_g_ = 1.66).

### Eukaryotes

In an ideal world of vertically inherited genes and infallible phylogeny, all genes would produce the same tree and all eukaryotic genes would trace to the same alphaproteobacterium (the mitochondrion) and the same are archaeon (host), plus the same cyanobacterium in the case of eukaryotes with plastids. But the real data from real genomes reveals that only a small minority of prokaryotic genes, much less than 1%, tend to be inherited vertically. How does the non-verticality of prokaryotic genes, genomes, and phyla impact our ability to infer the origin of eukaryotic genes? For all 3,420,731 protein coding genes from 150 eukaryotic genomes, we constructed clusters, merged them with their cognate prokaryotic clusters to generate eukaryote-prokaryote clusters (EPCs), constructed alignments and ML trees (see **Methods**). The red circles in **Fig 1** mark the prokaryotic clusters that were merged with their unique cognate eukaryotic clusters. The first question concerned eukaryote monophyly. There are many claims in the literature for LGT from prokaryotes to eukaryotes, but few are supported by prokaryotic reference samples that reflect the availability of genome data and fewer still, if any, are supported by systematic tests for eukaryote monophyly. Therefore, we looked closely at the possibility of LGT vs. eukaryote monophyly in our sample.

Among the 2,575 maximum-likelihood (ML) trees reconstructed from the merged eukaryote-prokaryote clusters, only 475 of the best trees found (18.4%) failed to recover eukaryotes as monophyletic. Does that finding represent evidence for LGT to eukaryotes in 18% of these trees, that is, is the best tree identified significantly better than the case of eukaryote monophyly? To test whether the lack of eukaryote monophyly in those 475 trees is due to reconstruction errors or due to prokaryote-eukaryote LGT, we compared the ML trees against trees with constrained eukaryote monophyly using likelihood tests. We employed the Kishino-Hasegawa test (KH), the approximately-unbiased test (AU) and the Shimodaira-Hasegawa test (SH) (see **Methods** for details). At the 5% significance level (p-value ≤ 0.05), the KH test rejected eukaryote monophyly for 6% of the trees (30 out of 475), that is, the null hypothesis (eukaryote monophyly) was rejected at a rate very close to that expected by chance. The AU test rejected eukaryote monophyly for 3 trees while the SH test did not reject eukaryote monophyly for any tree at the p-value of ≤ 0.05 (**S4 Fig** and **S5 Table**). Thus, the absence of a pure eukaryotic clade in some of the best trees found by ML trees results from challenges in distinguishing alternative trees that are statistically identical to the true tree, or to trees recovering eukaryote monophyly, in terms of their likelihood values, a problem that becomes more acute for phylogenetic inference using large samples because the tree space for the ML method to search grows exponentially. In terms of traits, eukaryotes are the strongest monophylum in nature, a status corroborated by the lack of any evidence that would support a case for the non-monophyly (LGT) of eukaryotic genes.

**Fig 4:**
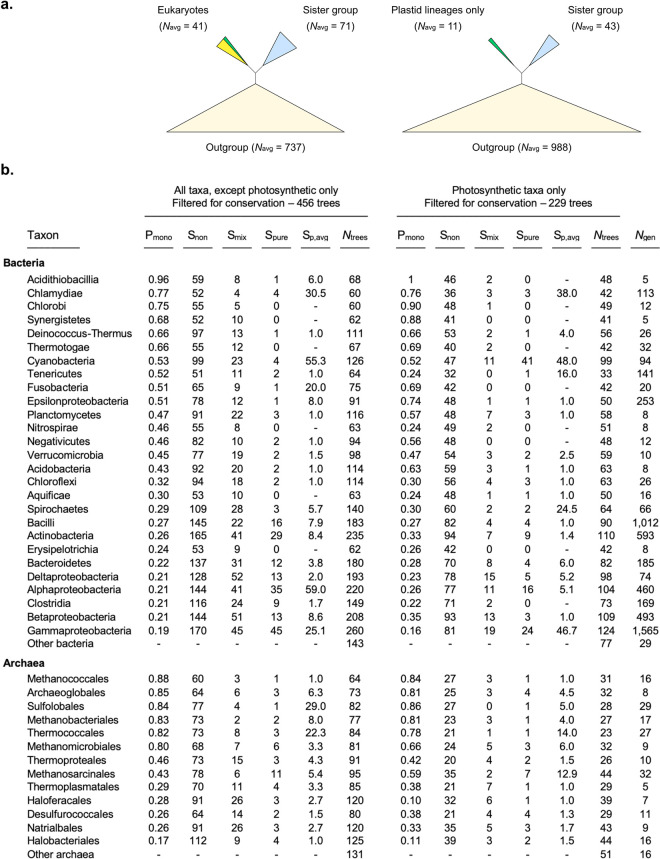
Identification of the prokaryotic sister group to the eukaryotes in 2,575 eukaryotic-prokaryotic unrooted gene trees (EPC). **a.** shows the average clade sizes for eukaryotes, the sister group to eukaryotes and the outgroup in the analyzed trees for (right) the 229 trees with only plastid derived lineages and (left) for the 456 EPCs containing all taxa except photosynthetic lineages. **b.** details the list of bacterial (top) and archaeal (bottom) phyla occurring in the trees with only plant lineages (right) and all other trees (left) that were filtered for conservation (average branch length of the tree ≤ 0.1). Archaeal and bacterial phyla with less than 5 representative species in the dataset were collapsed into ‘other archaea’ and ‘other bacteria’ groups. P_mono_ refers to the proportion of trees with a branch (split) separating the species of the respective phylum from all the others in the tree; S_non_ refers to the number of occurrence of the phylum only in the outgroup clade; S_mix_ refers to the number of occurrences of the phylum as a mixed sister (more than one phylum in the clade); S_pure_ refers to the number of occurrences of the phylum as pure sister (as the single phylum); S_p,avg_ shows the average size of the sister clade when the phylum occurs as a pure sister clade. *N*_trees_ show the number of occurrences of the phyla across the trees and *N*_gen_ indicates the number of species in each taxon included in the complete dataset.

What do trees say about the origin of eukaryotic genes? In the following, to avoid the effects of trees for poorly conserved genes (**Fig 1A**), we consider only those 685 trees in which the eukaryotic cluster mapped to one of the conserved prokaryotic clusters in **Fig 1B**. For each tree, we determined the prokaryotic sister group to the eukaryotic clade, and scored whether it was a pure sister containing sequences from only one prokaryotic phylum or a mixed sister group containing a mixed sister group from two or more phyla. The results are summarized in **Fig 4B**.

By the measure of phylogenetic inference, every prokaryotic phylum sampled in our study appears as a donor of genes to the eukaryote common ancestor, either by presence in a mixed sister group or as a pure sister (**Fig 4B**). This is true not only for bacteria, which would be expected to trace mitochondrial ancestry, but also for archaea, which since their discovery have been linked to the origin of the host. Can we naïvely interpret such observations at face value? Is it reasonable to believe that every phylum sampled here donated a gene, or several, to eukaryotes at their origin? If we break the data down to families, genera, or species, the number of donors grows accordingly (all prokaryotic organisms employed in this study were in the sister group to eukaryotes at least once), such that each gene in eukaryotes would correspond to an individual donation, as some would argue [54]. But that logic leads straight to the erroneous conclusion that ancestral plastid and mitochondrial genomes were assembled by acquisition *one gene at a time* [55] the converse of what they are in plain sight, namely reduced genomes of single bacterial endosymbionts [50] that underwent reductive evolution by transferring genes to the nucleus. Worse yet, the same problem ensues at the origin of plastids (**Fig 4B**, right column), because for photosynthetic eukaryotes again all phyla, including the archaea, appear as donors. Many genes that are germane to photosynthesis in eukaryotes trace to the plant common ancestor (plants being monophyletic) but only a minority of them trace specifically to Cyanobacteria, and those that do, do not trace to the same cyanobacterium [56,57].

If we only consider pure sister groups to eukaryotes, the most common apparent gene donor was Gammaproteobacteria, followed by Alphaproteobacteria, Actinobacteria and Bacilli. There is at least one theory in the literature invoking the participation of those groups at eukaryote origin [58]. However, a similar pattern recurs for plastids, which have the strongest pure sister signal from Cyanobacteria followed again by Gammaproteobacteria (for which there is no plastid origin theory) and Alphaproteobacteria. The problem of inferring symbionts from gene trees becomes more evident when we consider apparent archaeal contributions to the origins of plastids (**Fig 4B**), because there are no archaea that synthesize chlorophyll. We are confronted with a conflict. Blind inference of symbionts from trees cannot account for the origin of organelle genomes, the strongest form of evidence for the origin of organelles in the first place. The ‘one tree one donor’ logic carries a weighty premise that is never spelled out by its proponents, namely that the donated genes never underwent LGT among free living prokaryotes in the 1.5 billion years since organelle origin. If we approach the problem from the standpoint of theory testing in the presence of prior knowledge about the underlying process, namely one symbiont 1.5 billion years ago (as evidenced by the single origin of plastids and mitochondria respectively), what would look like many donors if we were to assume that prokaryotic gene evolution is vertical, is clearly the result of LGT among free-living prokaryotes, where, in real data, gene evolution is lateral.

**Fig 5:**
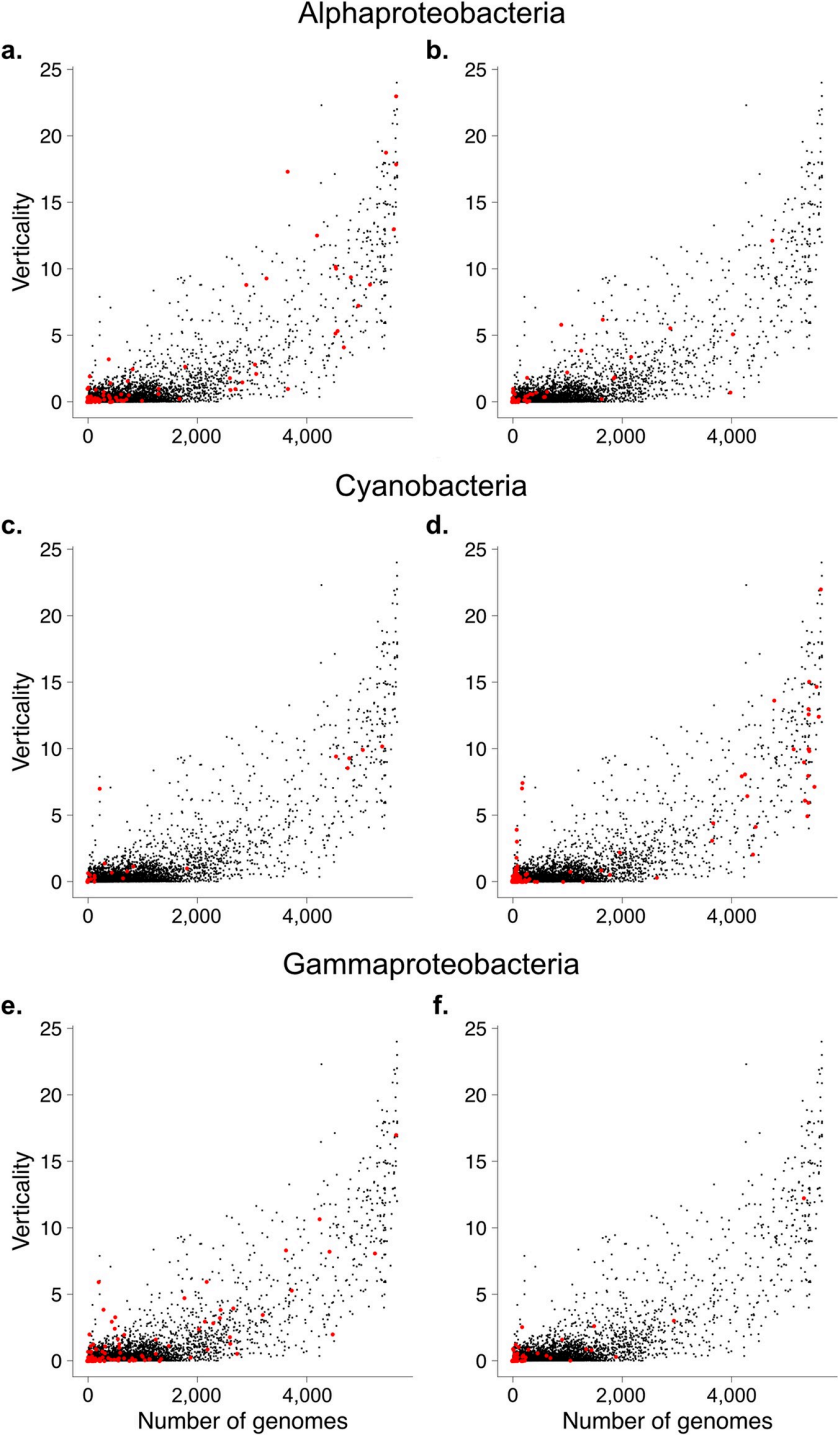
Mapping of EPCs to prokaryotic clusters. The EPCs were separated according to the pure sister group of eukaryotes in the unrooted trees: **a.** and **b.** Alphaproteobacteria, **c.** and **d.** Cyanobacteria, **e.** and **f.** Gammaproteobacteria. The left panel shows EPCs that may include all eukaryotic supergroups but no groups that include only photosynthetic lineages, the right panel shows only EPCs that only include photosynthetic eukaryotes (lineages from SAR, Hacrobia and Archaeplastida). Meaning the latter are indicative of plastid endosymbiosis. Plots for all taxa see **S5 Fig**.

For example, were the gammaproteobacterial signal in heterotrophic eukaryotes a result of gene acquisitions from donors with gammaproteobacterial rRNA, then that same signal would reflect a gammaproteobacterial origin of plastids (**Fig 4B**), which seems unlikely and is not covered by any theory. If on the other hand it were due to the low verticality of Gammaproteobacteria as a phylum, then Gammaproteobacteria should appear as the sister to many different groups of prokaryotes, which is precisely the observation (**Fig 3**). We asked whether there is a non-random signal across all genes that singles out Cyanobacteria (plastids) and Alphaproteobacteria (mitochondria) specifically as donors. This is shown in **Fig 5**, where we have plotted the distribution of trees that identify Alphaproteobacteria, Cyanobacteria or Gammaproteobacteria as pure sisters to (donors of) eukaryotic genes. Though Gammaproteobacteria appear as the pure sister in many trees (**Fig 4B**), the genes that do so are primarily of low verticality. Only the Alphaproteobacteria have a significant enrichment of vertical genes as sisters relative to the sample (**Fig 5A**), but the significance is marginal (p < 0.01). The Cyanobacteria are not significantly enriched in high verticality sisters, because of a large number of low verticality cases (**Fig 5C and 5D**). The majority of the gammaproteobacterial sister cases are low verticality genes (**Fig 5E and 5F**).

Throughout this discussion, we recall that the ancestor of mitochondria was not a phylum of proteobacteria, it was a single proteobacterium that engaged in a singular symbiosis. The same is true for plastids, whose origin was not the result of a symbiosis with the cyanobacterial phylum, it was a symbiosis with a single cyanobacterium. The genes that trace to those organelle origin events are, however, like almost all prokaryotic genes, of low verticality within prokaryotes.

A critic might ask whether eukaryotes, if their genes are of monophyletic origin relative to prokaryotes, score higher than all prokaryotes in terms of a comparable measure of verticality (supergroups instead of phyla). The problem there is a different one, namely paralogy. The underlying theme of eukaryotic genome evolution is recurrent gene and genome duplication [59,60], massive paralogy impairs eukaryote gene monophyly although gene duplications carry phylogenetic information in their own right [35]. The genes that have remained in plastid and mitochondrial genomes encode proteins involved in the electron transport chain of the bioenergetic membrane supporting photosynthesis and respiration, respectively, and the ribosomal proteins [61] involved in synthesizing those proteins in the organelle [62]. Why do those ribosomal proteins reflect an alphaproteobacterial [46] and cyanobacterial [56] origin of the organelle more clearly than non-ribosomal genes? It is not because non-ribosomal genes were acquired from different biological donors. Rather, it is because the prokaryotic reference set of ribosomal proteins is inherited in a vertical manner among free living prokaryotes; all other prokaryotic genes are inherited more laterally (**Fig 1**), evoking the illusion of many different donors to eukaryotes in phylogenetic analyses (**Fig 4B**). Yet that illusion rests upon the tacit assumption that prokaryotes inherit their genes vertically, which is however untrue [2,34,63,64,65].

## Discussion

Even though gene evolution in prokaryotes has substantial lateral components, rRNA-based investigations and some protein phylogenetic studies tend to recover groups that microbiologists recognized long before molecular systematics. Hence the groups are in some cases real and there must be a vertical component to prokaryote evolution. The vertical component has, however, been difficult to quantify across lineages. Equally elusive have been estimates for verticality itself, yet suitable methods to quantify that component have been obscure, as have means to quantify verticality across prokaryotic genes. Quantification of discordance in tree comparisons represents one approach [66] to estimate LGT or lack thereof, but its utility is limited when large genome samples are involved, because the number of possible trees exceeds the number that a computer can examine by hundreds of orders of magnitude for trees containing 60 leaves or more. By exploiting the common wisdom that phylogeny works better at the tips of trees than at their deeper branches, we have obtained robust estimates of verticality.

Though many genes that are currently used in molecular systematic studies based on their widespread occurrence have low verticality, across all genes ***V*** does increase with distribution density. We suggest that this is so because the displacement of a well-regulated preexisting copy is less likely than the transient and rarely permanent, in some cases lineage founding [67], acquisition of novel traits. That most genes in prokaryotes have both restricted distribution and low verticality underscores the need to identify genes that are inherited vertically across large data sets for the purpose of higher-level broad scale phylogenetic analyses. We found no genes among the 101,422 total clusters and 8,547 conserved clusters that recovered monophyly of all 40 phyla. At the same time all phyla were disguised as gene donors to eukaryotes both at the origin of mitochondria and at the origin of plastids because of LGT among the prokaryotic reference set.

The spectrum of verticality across genes observed here precludes the need to propose, based on trees that implicate non-alphaproteobacterial or non-cyanobacterial gene donors, genetic contributors at the origin of eukaryotes beyond the host, the mitochondrion and, later, the cyanobacterial antecedent of plastids, because LGT among prokaryotes can account for the same gene-tree based observations, more directly and with fewer corollary assumptions, while simultaneously accounting for a larger set of observations among the prokaryotic reference set. The criterion of verticality can furthermore be of practical use in the selection of genes for molecular systematic studies.

## Methods

### Prokaryotic dataset

Protein sequences for 5,655 prokaryotic genomes were downloaded from NCBI [68] (version September 2016; see **S3 Table** for detailed species composition). We performed all-vs-all BLAST [69] searches (BlastP version 2.5.0 with default parameters) and selected all reciprocal best hits with e-value ≤ 10^−10^. The protein pairs were aligned with the Needleman-Wunsch algorithm [70] (EMBOSS needle) and the pairs with global identity values < 25% were discarded. The retained global identity pairs were used for clustering using Markov clustering algorithm [71] (MCL) version 12–068, changing default parameters for pruning (-P 180000, -S 19800, -R 25200). Clusters distributed in at least 4 genomes spanning 2 prokaryotic phyla were retained, resulting in 101,422 used clusters in total. Sequence alignments for each cluster were generated using MAFFT [72], with the iterative refinement method that incorporates local pairwise alignment information (L-INS-i; version 7.130). The resulting alignments were used to reconstruct maximum-likelihood trees with RAxML version 8.2.8 [73] (parameters: -m PROTCATWAG -p 12345) (**S9 Table**). The trees were rooted with the Minimal Ancestor Deviation method (MAD) [74].

### Eukaryotic dataset

Protein sequences for 150 eukaryotic genomes were downloaded from NCBI, Ensembl Protists and JGI (see **S7 Table** for detailed species composition). To construct gene families, we performed an all-vs-all BLAST [66] of the eukaryotic proteins (BlastP version 2.5.0 with default parameters) and selected the reciprocal best BLAST hits with e-value ≤ 10^−10^. The protein pairs were aligned with the Needleman-Wunsch algorithm (EMBOSS needle) [70] and the pairs with global identity values < 25% were discarded. The retained global identity pairs were used to construct gene families with the MCL algorithm [71] (version 12–068) with default parameters. We considered only the gene families with multiple gene copies in at least two eukaryotic genomes. Protein-sequence alignments for the multi-copy gene families were generated using MAFFT [72], with the iterative refinement method that incorporates local pairwise alignment information (L-INS-i, version 7.130). The alignments were used to reconstruct maximum likelihood trees with IQ-tree [75], applying the parameters ‘-bb 1000’ and ‘-alrt 1000’ (version 1.6.5), with subsequent rooting with MAD [74].

### Eukaryotic-prokaryotic dataset

To assemble a dataset of conserved genes for phylogenies linking prokaryotes and eukaryotes, eukaryotic, archaeal and bacterial protein sequences were first clustered separately before homologous clusters between eukaryotes and prokaryotes were identified. Eukaryotic protein sequences from 150 genomes (**S7 Table**) were clustered with MCL [71] using global identities from best reciprocal BLAST hits for protein pairs with e-value ≤ 10^−10^ and global identity ≥ 40%. The clusters with genes distributed in at least two eukaryotic genomes were retained. Similarly, prokaryotic protein sequences from 5,655 genomes were clustered using the best reciprocal BLAST for protein pairs with e-value ≤ 10^−10^ and global identity ≥ 25% (for archaea and bacteria, separately). The resulting clusters with gene copies in at least 5 prokaryotic genomes were retained. Eukaryotic and prokaryotic clusters were merged using the reciprocal best cluster procedure [57]. We merged a eukaryotic cluster with a prokaryotic cluster if ≥ 50% of the eukaryotic sequences in the cluster have their best reciprocal BLAST hit in the same prokaryotic cluster and vice-versa (cut-offs: e-value ≤ 10^−10^ and local identity ≥ 30%) yielding 2,587 eukaryotic-prokaryotic clusters (EPCs). EPCs with ambiguous cluster assignment were discarded. Protein-sequence alignments for 2,575 EPCs were generated using MAFFT (L-INS-i, version 7.130); for twelve clusters, the alignment did not compute as sequence quality was low. The alignments were used to reconstruct maximum-likelihood trees with IQ-tree (version 1.6.5) employing the parameters ‘-bb 1000’ and ‘-alrt 1000’ (**S5 Table**).

### Verticality

The verticality measure for each gene was defined as the sum of monophyly scores for all monophyletic taxa present in the unrooted trees. Only for the calculation of the average root-to-tip measurements (**S2 Fig)** rooted trees were necessary. This supplementary analysis was then performed with MAD rooted trees. Our species set contains 42 taxa corresponding mostly to phyla level, except for Proteobacteria, Firmicutes and Achaea (see **S8 Table**). For a given tree, the monophyly score *S*_*a*_ for taxon *a* was defined as:

Sa=naNa,ifaismonophyleticintree

$$S_{a}=0,\;otherwise$$

where *n*_*a*_ is the number of species in the tree affiliated to *a* and *N*_*a*_ is the total number of species from *a* among the 5,655 genomes in our set. The verticality measure *V*_*g*_ for a gene was then defined as:

$$V_{g}=\sum S_{a},\;for\;all\;taxa\;a\;present\;in\;tree$$

The analyses were conducted with custom scripts using NewickUtilities [76] and ETE [77]. Taxon and genome verticality were defined as the average gene verticality across all gene trees where the taxon (or genome) were present. In addition, weighted taxon verticality for each taxon was defined as the weighted average across all gene trees where the phylum appears, weighted meaning here that values of monophyletic clusters were summed up while values of paraphyletic clusters were subtracted.

### Functional annotation

Two annotation strategies were performed for each protein cluster. First, protein annotation information according to the BRITE (Biomolecular Reaction pathways for Information Transfer and Expression) hierarchy was downloaded from the Kyoto Encyclopedia of Genes and Genomes (KEGG v. September 2017) website [78], including protein sequences and their assigned function according to the KO numbers. The protein sequences of the 5,655 organisms were mapped to the KEGG database using local alignments with ‘blastp’. Only the best BLAST hit of the given protein with an e-value ≤ 10^−10^ and alignment coverage of 80% was selected. After assigning a function based on the KO numbers of KEGG for each protein in the clusters, the majority rule was applied to identify the function for each cluster. The occurrence of the function of each protein in the cluster was added and the most prevalent function was assigned for each cluster.

The second annotation used the NCBI headers. For this, the appearance of a word among all sequence headers of a cluster was counted. Then, each header was given a score based on the sum of how often its words appeared among all headers. The header with the highest score was then chosen as the cluster annotation.

### Tests for eukaryote monophyly

For 475 gene trees where eukaryotes were not recovered as monophyletic, we conducted the Kishino-Hasegawa (KH) test [79], the Shimodaira-Hasegawa (SH) test [80] and the approximately-unbiased (AU) test [81] to assess whether the observed non-monophyly was statistically significant. We reconstructed trees constraining eukaryotic sequences to be monophyletic, but not imposing any other topological constraint, using FastTree [82] (version 2.1.10 SSE3) and recording all trees explored during the tree search with the ‘-log’ parameter. The sample of monophyletic trees were used as input in IQ-tree (version 2.0.3; parameter: ‘-zb 100000 -au’) to perform the KH, SH and AU tests against the unconstrained tree (non-monophyletic). If the best constrained tree did not show significant difference relative to the unconstrained tree (p-value ≤ 0.05), then we considered that eukaryotic monophyly cannot be rejected.

### Sister analyses

#### Prokaryotes

The sister for each prokaryotic taxon was defined as the clade with the smallest branch to the query clade. Two cases had to be differentiated: Mono- or paraphyletic taxonomic groups in a tree. Monophyly was tested as described above with NewickUtilities. For these taxonomic groups, the sister groups could also be directly obtained by using NewickUtilities (nw_clade -s). Finally, all different taxonomic groups in the sister groups were given a score equal to their proportion in the sister group. For paraphyly of a taxonomic group (main group), the monophyletic subgroups were determined with the python package ETE 3 [77]. Each of these subgroups was handled as an individual group in the cluster and the sister clades were determined. Again, if several taxonomic groups were present in a sister group, then these were given a score equal to their proportion in the sister. To get from the scores of each subgroup to the total score of the main group, each subgroup´s scores was multiplied by the proportion of genomes the subgroup has of the main group. Subsequently, the score of a potential sister group to the main group was calculated by summing up its adjusted score over all subgroups. For each taxonomic group, sister scores were normalized by dividing each score through the highest sister score and then plotted as a heatmap.

#### Eukaryotes

To infer the prokaryotic sisters to eukaryotes we used 2,575 EPC trees. The majority of the EPC trees (2,100) support eukaryotic monophyly. For 475 trees for which eukaryotes did not branch together we recalculated trees constraining eukaryotic monophyly because the Shimodaira-Hasegawa tests failed to reject eukaryotic monophyly for all the 475 trees (see **Methods** section ‘tests for eukaryote monophyly’ and main text). Note that in unrooted trees for which eukaryotes are monophyletic, the prokaryotic side of the tree is bisected by one internal node into two prokaryotic subclades, each subclade being the potential sister to eukaryotes (**Fig 4A**). We considered the prokaryotic subclade with the smallest number of leaves for our inferences of sister-relations.

### Terminal gene duplications

Terminal gene duplications were inferred using the rooted gene trees as pairs of genes sampled from the same genome that appeared as reciprocal sisters in the tree. Gene trees with ambiguous MAD roots were discarded.

### Statistical tests

To test the correlations of variables, the Pearson´s correlation test was used [83]. The test results of various combinations for example Number of genomes and number of phyla, that are not mentioned in the text are given in **S2 Table**.

## Supporting information

S1 Table All relevant information about all 101,422 clusters employed in this study. (XLSX)

S2 Table Calculated correlations for Fig 1 and S1 Fig. (TIF)

S3 Table List of all prokaryotic organisms. (TXT)

S4 Table Average verticality per genome and per taxonomic group (phylum). (XLSX)

S5 Table List of all 2,575 EPC trees with information if likelihood ratio test was performed. (XLSX)

S6 Table Identity and Annotation of the 100 most vertical clusters. (XLSX)

S7 Table List of all eukaryotic organisms. (TXT)

S8 Table List of all 42 taxonomic groups with labels. (TXT)

S9 Table List of all 101,422 RAxML-MAD rooted prokaryote-only trees employed in this analysis. (DOCX)

S10 Table Underlying data for S2 Fig. (XLSX)

S1 Fig Cumulative distribution function of the fraction of terminal duplicates normalized for genome size compared to the distributions in eukaryotes versus prokaryotes using all genes. **a.** Shows the cumulative frequency of the proportion of duplications of all 5,655 prokaryotic organisms (red) compared to the 150 eukaryotes (blue) in our dataset. **b.** Shows the cumulative frequency of 100 random sample sets of 150 prokaryotic organisms each (red) versus the 150 eukaryotic organisms (blue) in the dataset.
(TIF)

S2 Fig Relationship of Verticality, calculated from average root-leave distance in MAD rooted trees, and number of genomes in cluster. Comparison of verticality, normalized by multiplying raw monophyly count by their average root to leave distance of each tree, and number of genomes in a protein cluster for **a.** all clusters (n = 101,422) and **b.** all conserved clusters (average branch length ≥ 0.1; n = 8,547). The plot is created analogous to Fig 1 in the main text and this alternative verticality calculation also correlates to number of genomes (A: p < 10–300, Pearson´s R2 = 0.571; B: p < 10–300, R2 = 0.686). The correlation is more consistent when comparing verticality to number of phyla represented in a cluster (a: p < 10–300, Pearson´s R2 = 0.754; b: p < 10–300, R2 = 0.767, see S2 Table for more details). The eukaryotic-prokaryotic clusters (EPCs) are highlighted in red and the clusters that correspond to a gene from the mitochondrial genome of *Reclinomonas americana* [45] are displayed in blue triangles along the abscissa of the plot and in the graph. For the latter, the gene identifier was noted above each plot. Ribosomal proteins are indicated by the black diamond on the right of each plot and in the graph [6]. Notably, these clusters show a steep decline in clusters with lower verticality among the conserved clusters.
(TIF)

S3 Fig Schematic representation of the calculation for the verticality of a gene (Vg) on the base of one tree with 30 genomes spanning four phyla. Each phylum is indicated by one color as depicted in the legend of the table. If the phylum is monophyletic in the tree, the number of genomes in the tree are divided by the number of genomes of this phylum present in the dataset of 5,655 organisms–phyla **e** and **f** in the panels **a.** and **b.** of the figure. If the phylum is paraphyletic, the verticality is set to '0'–phyla **g** and **h** in panels **c.** and **d.** of the figure. This number represents the verticality for each phylum. The sum of all verticality scores for the phyla in the tree is then the verticality for the tree and conversely, for the gene.
(TIF)

S4 Fig Likelihood tests of eukaryote monophyly. The Kishino-Hasegawa (KH) test, Shimodaira-Hasegawa (SH) test and the Approximately-Unbiased (AU) test were performed for 475 prokaryote-eukaryote genes for which eukaryotes were not recovered monophyletic in the ML trees. The histogram shows the distribution of p-values (horizontal axis) for the tests of the unconstrained ML trees against ML trees with constrained eukaryote monophyly. A test was considered significant (eukaryote monophyly was rejected) if p-value ≤ 0.05.
(TIF)

S5 Fig EPCs with pure sister taxon mapped to conserved clusters. Mapping of EPCs to prokaryotic clusters. The EPCs were separated according to the pure sister group of eukaryotes in the trees and plotted in the same way as in Fig 4 of the main text. The left panel shows EPCs that may include all eukaryotic supergroups, the right panel shows only EPCs that include archaeplastidal eukaryotes. Meaning the latter are indicative of plastid endosymbiosis. For a better overview a headline is included in each plot that lists the taxonomic group represented, if it shows EPCs linked to the mitochondrial (‘P and O’, left panel) or to the plastidal endosymbiosis event (‘Plant only’, right panel), and the number of EPCs that are shown as red dots.
(GZ) Click here for additional data file.
