# Supplementary figures and images for: A spectrum of verticality across genes
Source: PLoS Genet. 2020 Nov 2;16(11):e1009200. doi: 10.1371/journal.pgen.1009200 (PMC7660906; doi:10.1371/journal.pgen.1009200)

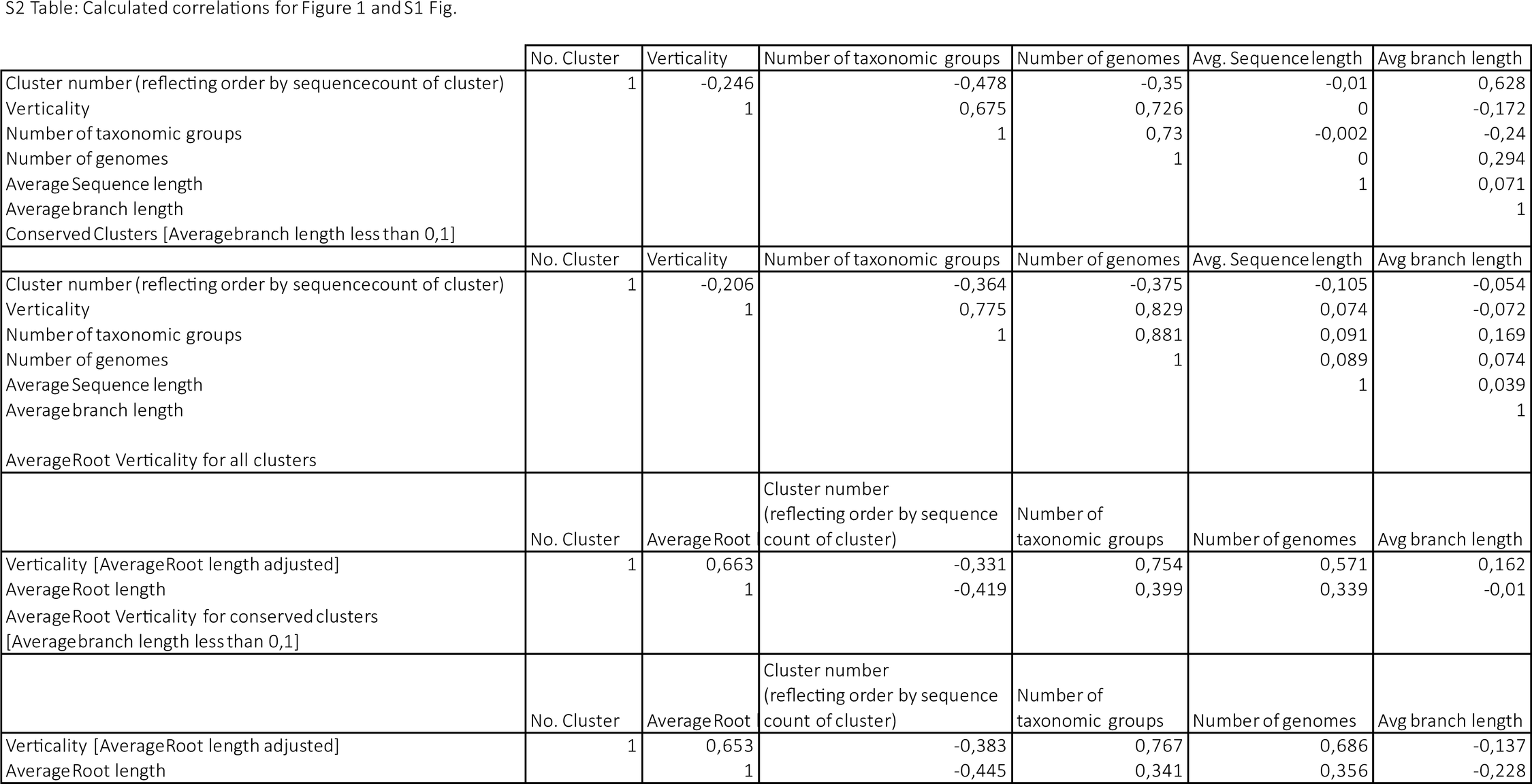

Supplement: S2 Table — (TIF) [file pgen.1009200.s002.tif]

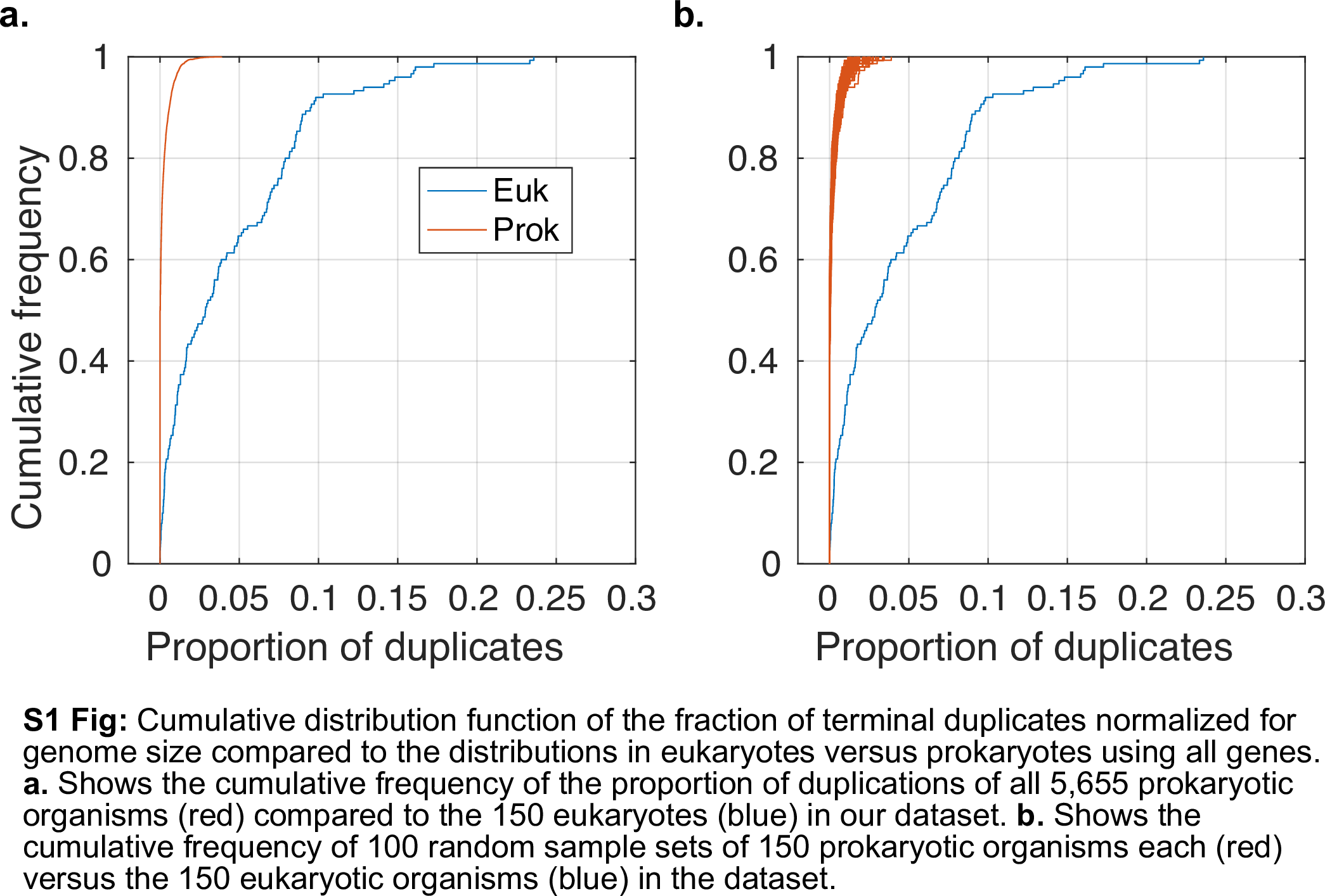

Supplement: S1 Fig — a. Shows the cumulative frequency of the proportion of duplications of all 5,655 prokaryotic organisms (red) compared to the 150 eukaryotes (blue) in our dataset. b. Shows the cumulative frequency of 100 random sample sets of 150 prokaryotic organisms each (red) versus the 150 eukaryotic organisms (blue) in the dataset. (TIF) [file pgen.1009200.s011.tif]

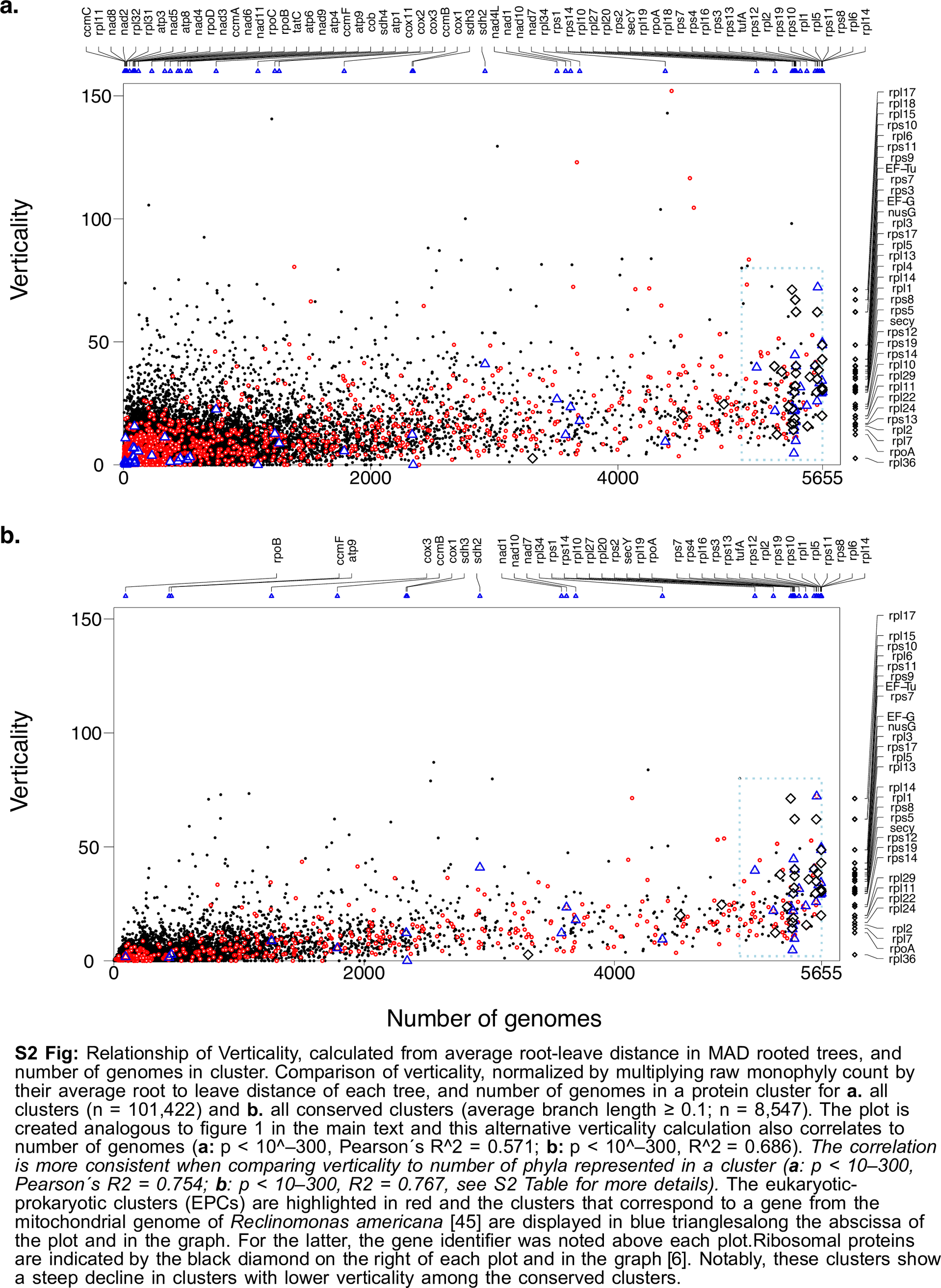

Supplement: S2 Fig — Comparison of verticality, normalized by multiplying raw monophyly count by their average root to leave distance of each tree, and number of genomes in a protein cluster for a. all clusters (n = 101,422) and b. all conserved clusters (average branch length ≥ 0.1; n = 8,547). The plot is created analogous to Fig 1 in the main text and this alternative verticality calculation also correlates to number of genomes (A: p < 10–300, Pearson´s R2 = 0.571; B: p < 10–300, R2 = 0.686). The correlation is more consistent when comparing verticality to number of phyla represented in a cluster (a: p < 10–300, Pearson´s R2 = 0.754; b: p < 10–300, R2 = 0.767, see S2 Table for more details). The eukaryotic-prokaryotic clusters (EPCs) are highlighted in red and the clusters that correspond to a gene from the mitochondrial genome of Reclinomonas americana [45] are displayed in blue triangles along the abscissa of the plot and in the graph. For the latter, the gene identifier was noted above each plot. Ribosomal proteins are indicated by the black diamond on the right of each plot and in the graph [6]. Notably, these clusters show a steep decline in clusters with lower verticality among the conserved clusters. (TIF) [file pgen.1009200.s012.tif]

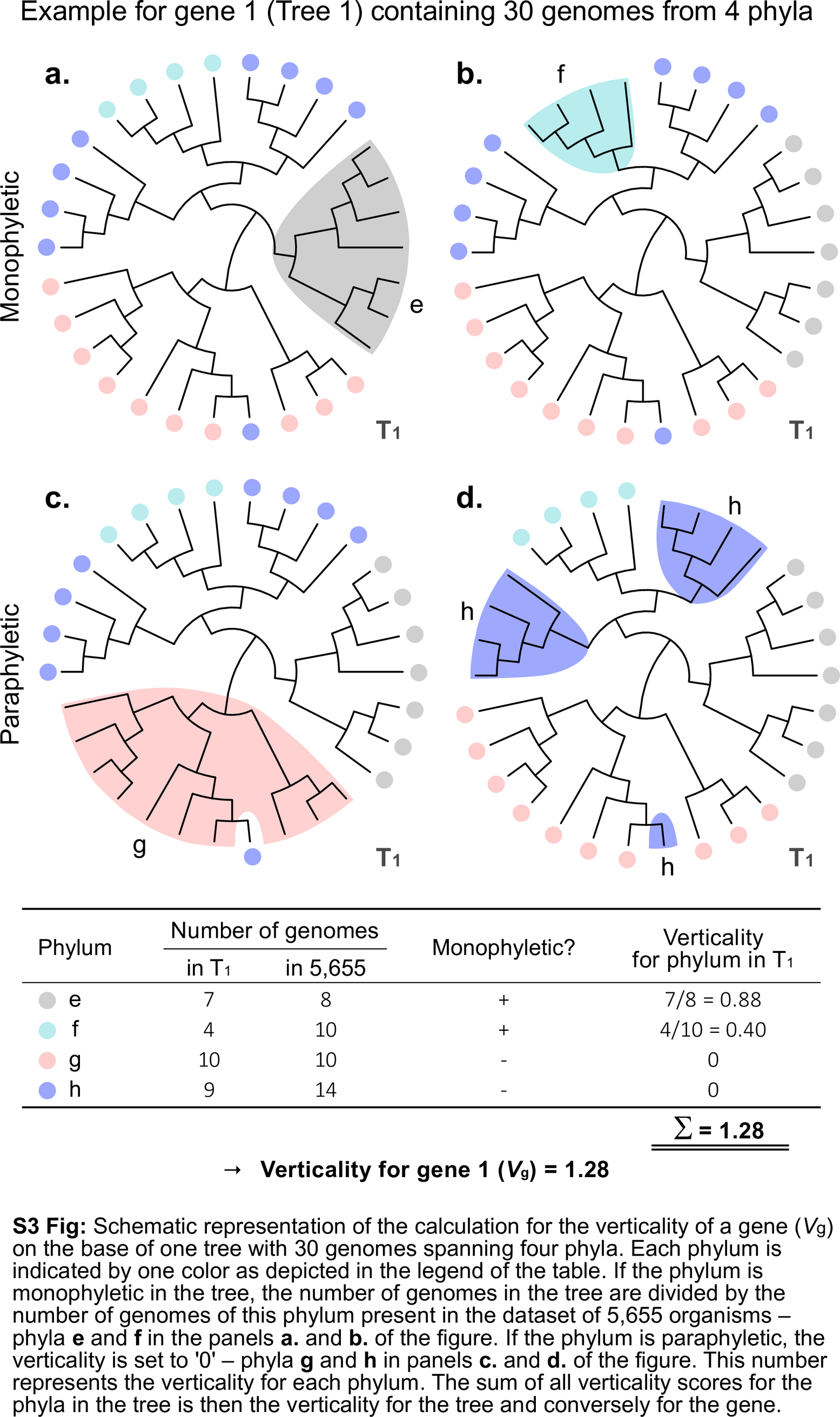

Supplement: S3 Fig — Each phylum is indicated by one color as depicted in the legend of the table. If the phylum is monophyletic in the tree, the number of genomes in the tree are divided by the number of genomes of this phylum present in the dataset of 5,655 organisms–phyla e and f in the panels a. and b. of the figure. If the phylum is paraphyletic, the verticality is set to '0'–phyla g and h in panels c. and d. of the figure. This number represents the verticality for each phylum. The sum of all verticality scores for the phyla in the tree is then the verticality for the tree and conversely, for the gene. (TIF) [file pgen.1009200.s013.tif]

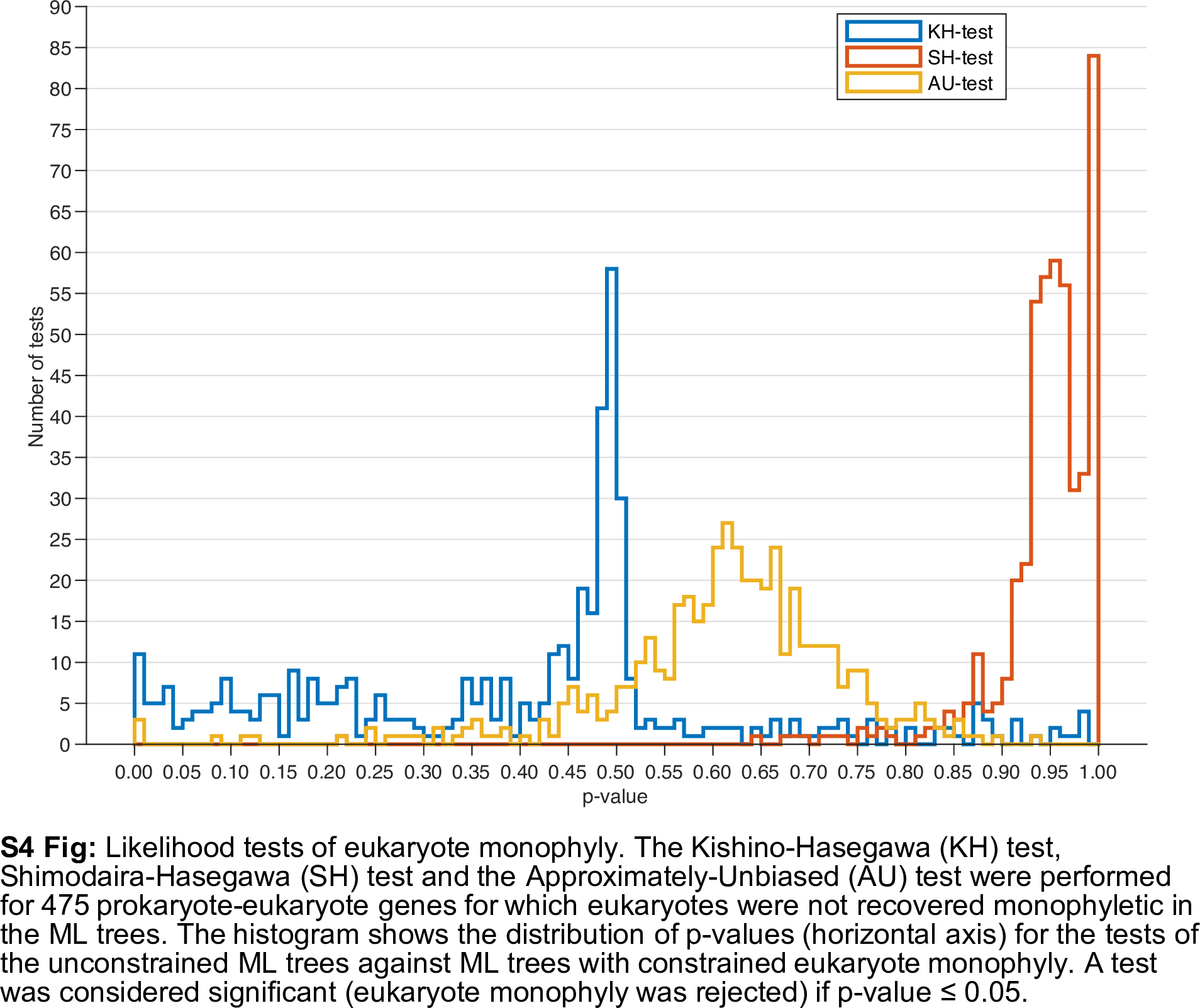

Supplement: S4 Fig — The Kishino-Hasegawa (KH) test, Shimodaira-Hasegawa (SH) test and the Approximately-Unbiased (AU) test were performed for 475 prokaryote-eukaryote genes for which eukaryotes were not recovered monophyletic in the ML trees. The histogram shows the distribution of p-values (horizontal axis) for the tests of the unconstrained ML trees against ML trees with constrained eukaryote monophyly. A test was considered significant (eukaryote monophyly was rejected) if p-value ≤ 0.05. (TIF) [file pgen.1009200.s014.tif]

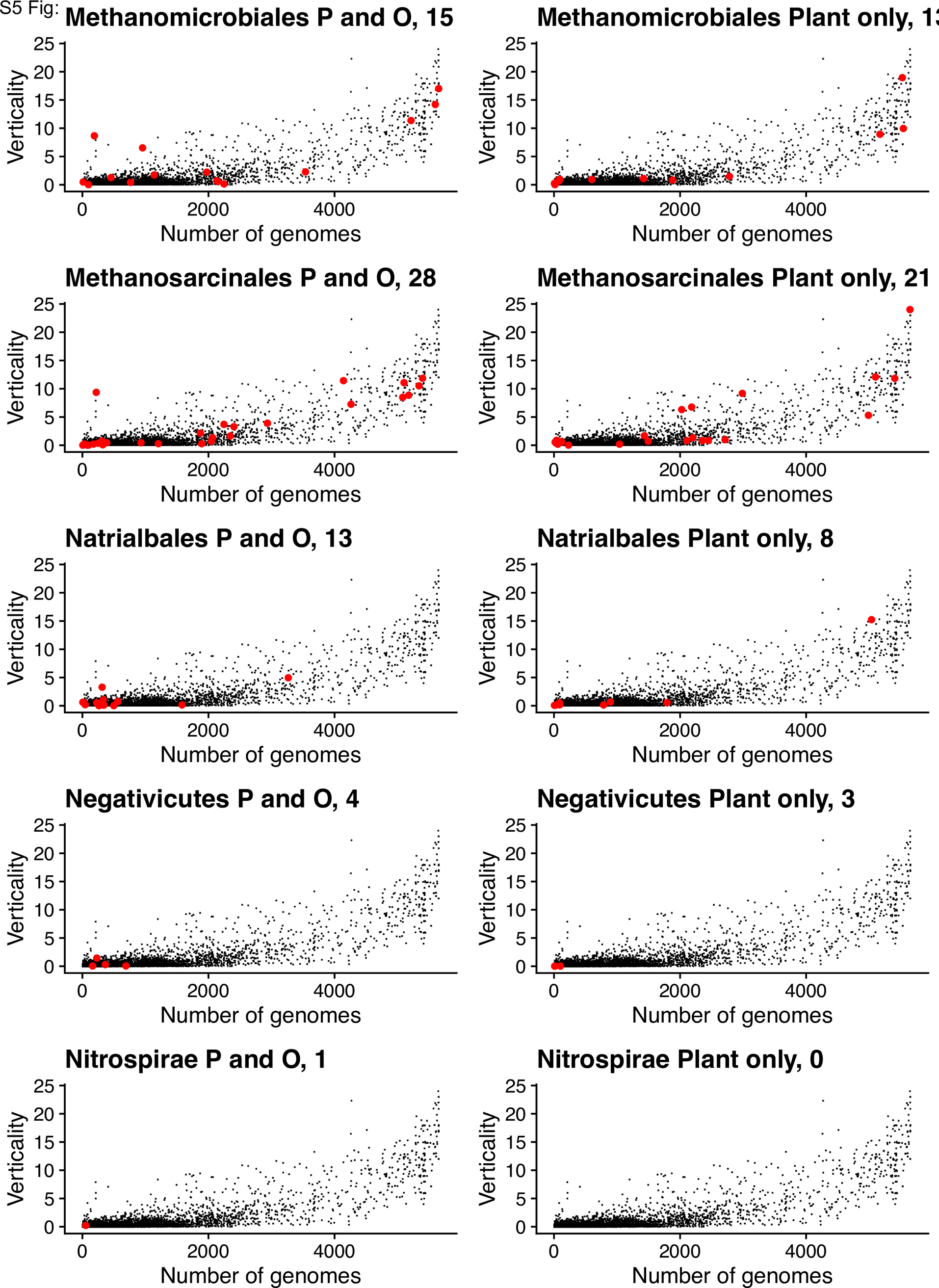

Supplement: S5 Fig — Mapping of EPCs to prokaryotic clusters. The EPCs were separated according to the pure sister group of eukaryotes in the trees and plotted in the same way as in Fig 4 of the main text. The left panel shows EPCs that may include all eukaryotic supergroups, the right panel shows only EPCs that include archaeplastidal eukaryotes. Meaning the latter are indicative of plastid endosymbiosis. For a better overview a headline is included in each plot that lists the taxonomic group represented, if it shows EPCs linked to the mitochondrial (‘P and O’, left panel) or to the plastidal endosymbiosis event (‘Plant only’, right panel), and the number of EPCs that are shown as red dots. (GZ) [file pgen.1009200.s015.tar.gz › S5_Fig/S5.6_Fig.tif]

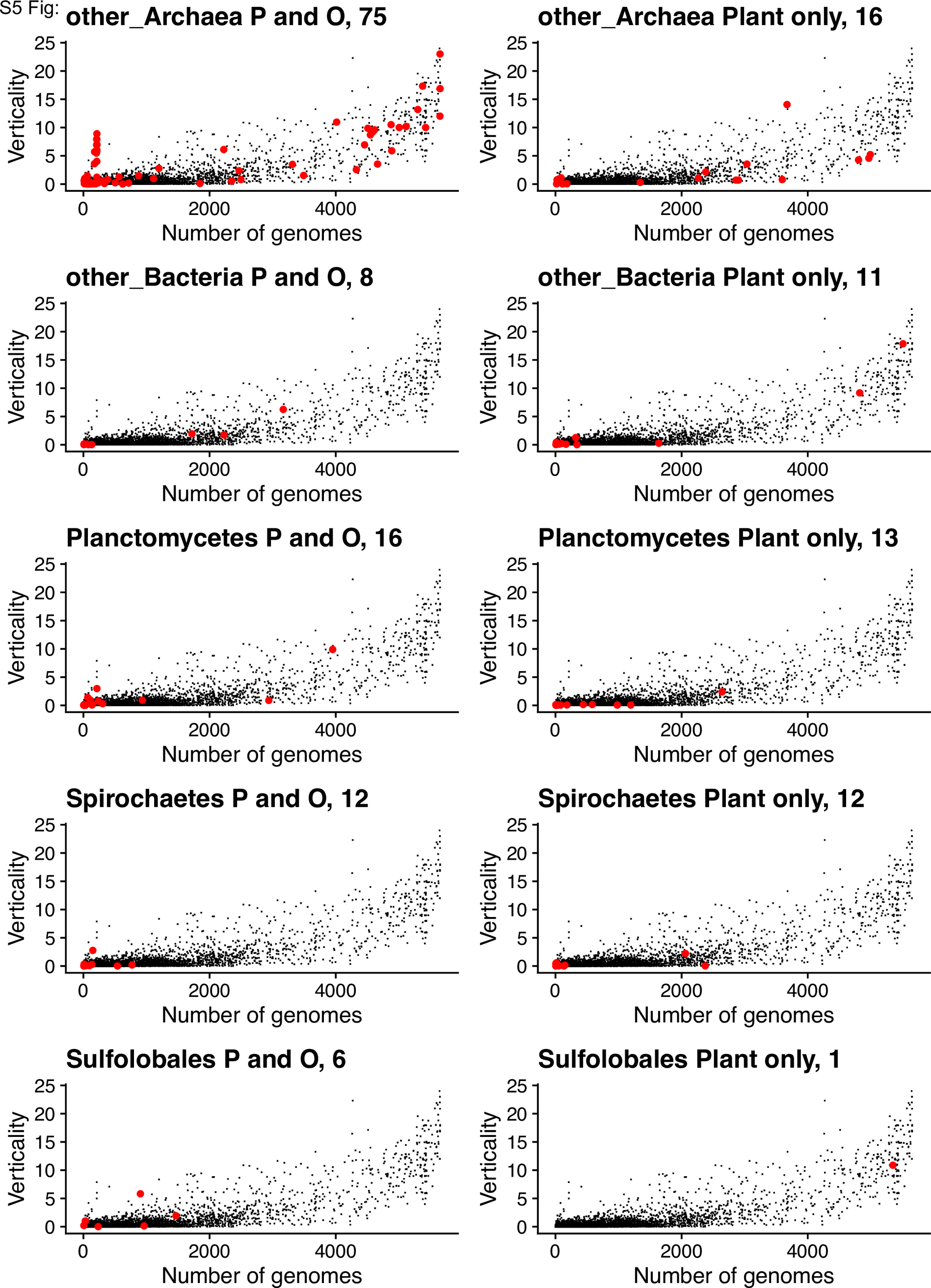

Supplement: S5 Fig — Mapping of EPCs to prokaryotic clusters. The EPCs were separated according to the pure sister group of eukaryotes in the trees and plotted in the same way as in Fig 4 of the main text. The left panel shows EPCs that may include all eukaryotic supergroups, the right panel shows only EPCs that include archaeplastidal eukaryotes. Meaning the latter are indicative of plastid endosymbiosis. For a better overview a headline is included in each plot that lists the taxonomic group represented, if it shows EPCs linked to the mitochondrial (‘P and O’, left panel) or to the plastidal endosymbiosis event (‘Plant only’, right panel), and the number of EPCs that are shown as red dots. (GZ) [file pgen.1009200.s015.tar.gz › S5_Fig/S5.7_Fig.tif]

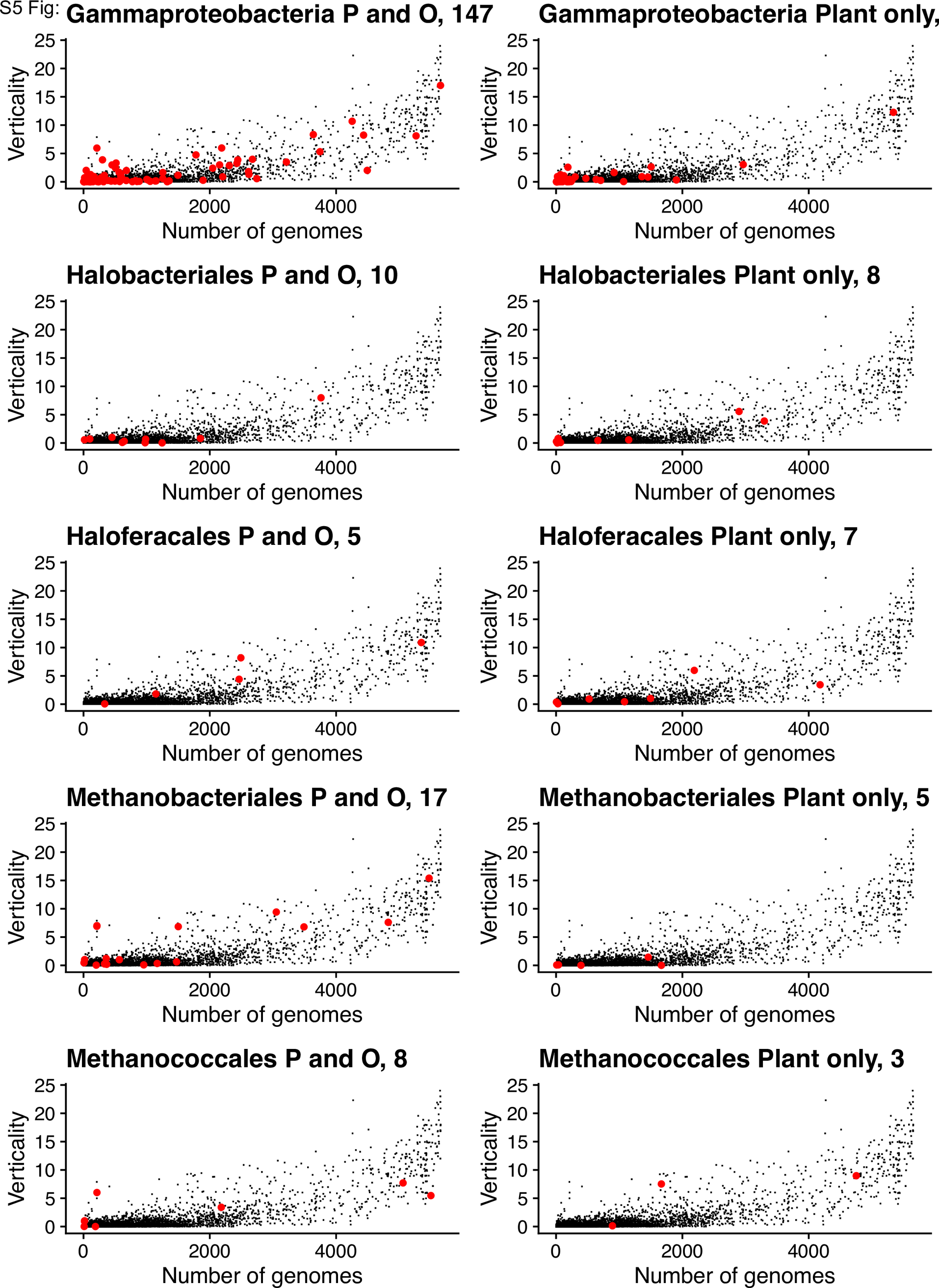

Supplement: S5 Fig — Mapping of EPCs to prokaryotic clusters. The EPCs were separated according to the pure sister group of eukaryotes in the trees and plotted in the same way as in Fig 4 of the main text. The left panel shows EPCs that may include all eukaryotic supergroups, the right panel shows only EPCs that include archaeplastidal eukaryotes. Meaning the latter are indicative of plastid endosymbiosis. For a better overview a headline is included in each plot that lists the taxonomic group represented, if it shows EPCs linked to the mitochondrial (‘P and O’, left panel) or to the plastidal endosymbiosis event (‘Plant only’, right panel), and the number of EPCs that are shown as red dots. (GZ) [file pgen.1009200.s015.tar.gz › S5_Fig/S5.5_Fig.tif]

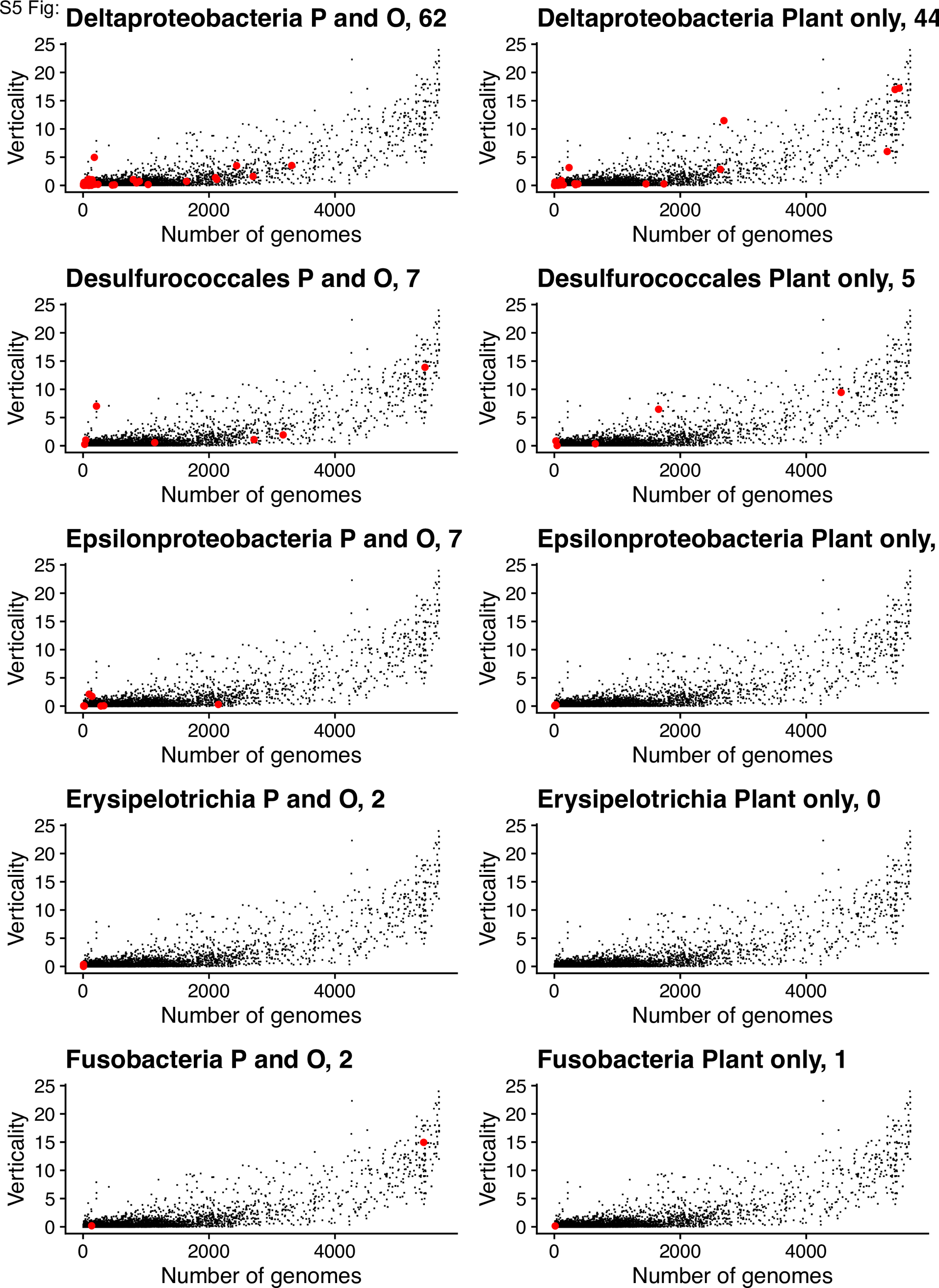

Supplement: S5 Fig — Mapping of EPCs to prokaryotic clusters. The EPCs were separated according to the pure sister group of eukaryotes in the trees and plotted in the same way as in Fig 4 of the main text. The left panel shows EPCs that may include all eukaryotic supergroups, the right panel shows only EPCs that include archaeplastidal eukaryotes. Meaning the latter are indicative of plastid endosymbiosis. For a better overview a headline is included in each plot that lists the taxonomic group represented, if it shows EPCs linked to the mitochondrial (‘P and O’, left panel) or to the plastidal endosymbiosis event (‘Plant only’, right panel), and the number of EPCs that are shown as red dots. (GZ) [file pgen.1009200.s015.tar.gz › S5_Fig/S5.4_Fig.tif]

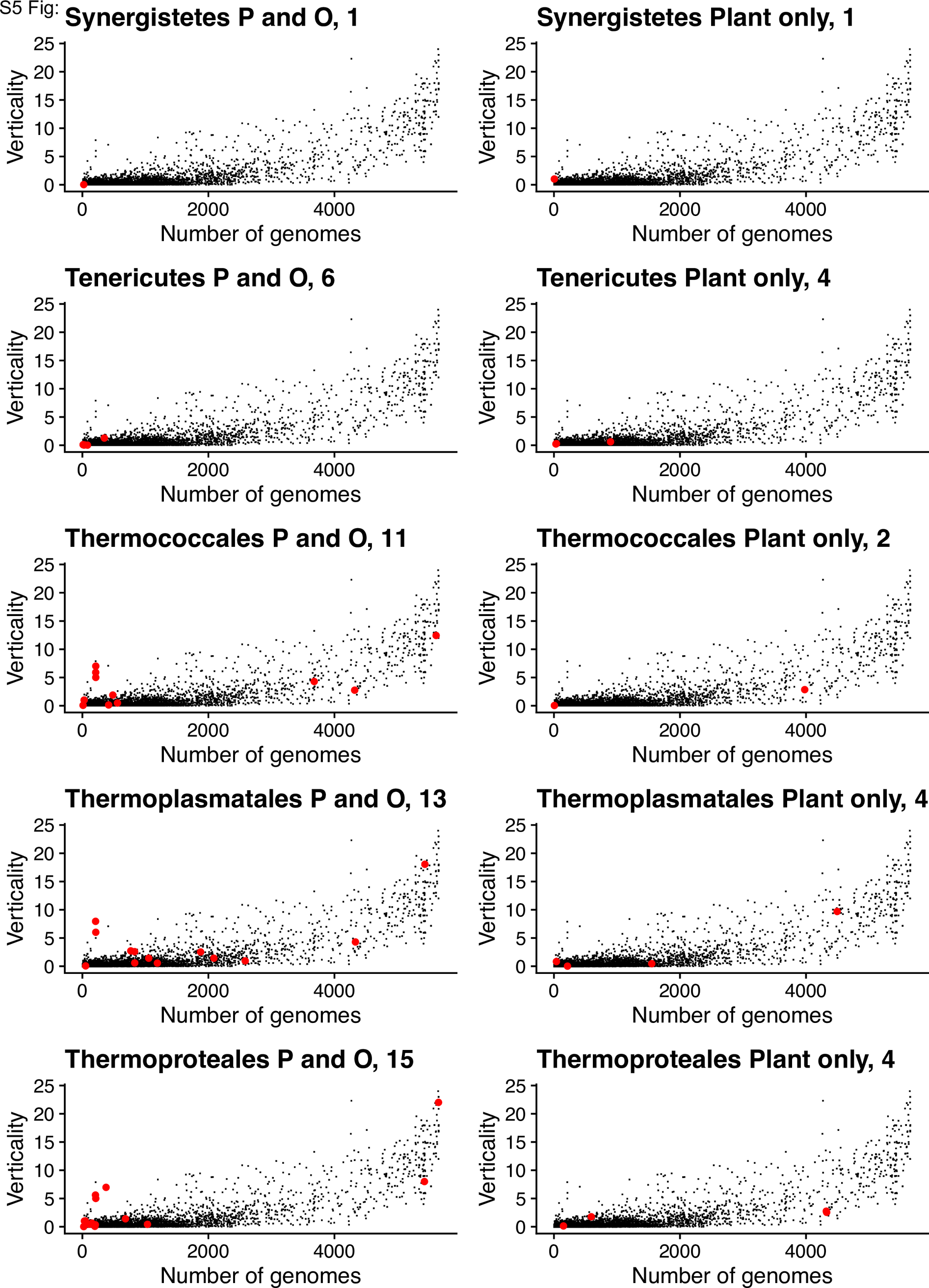

Supplement: S5 Fig — Mapping of EPCs to prokaryotic clusters. The EPCs were separated according to the pure sister group of eukaryotes in the trees and plotted in the same way as in Fig 4 of the main text. The left panel shows EPCs that may include all eukaryotic supergroups, the right panel shows only EPCs that include archaeplastidal eukaryotes. Meaning the latter are indicative of plastid endosymbiosis. For a better overview a headline is included in each plot that lists the taxonomic group represented, if it shows EPCs linked to the mitochondrial (‘P and O’, left panel) or to the plastidal endosymbiosis event (‘Plant only’, right panel), and the number of EPCs that are shown as red dots. (GZ) [file pgen.1009200.s015.tar.gz › S5_Fig/S5.8_Fig.tif]

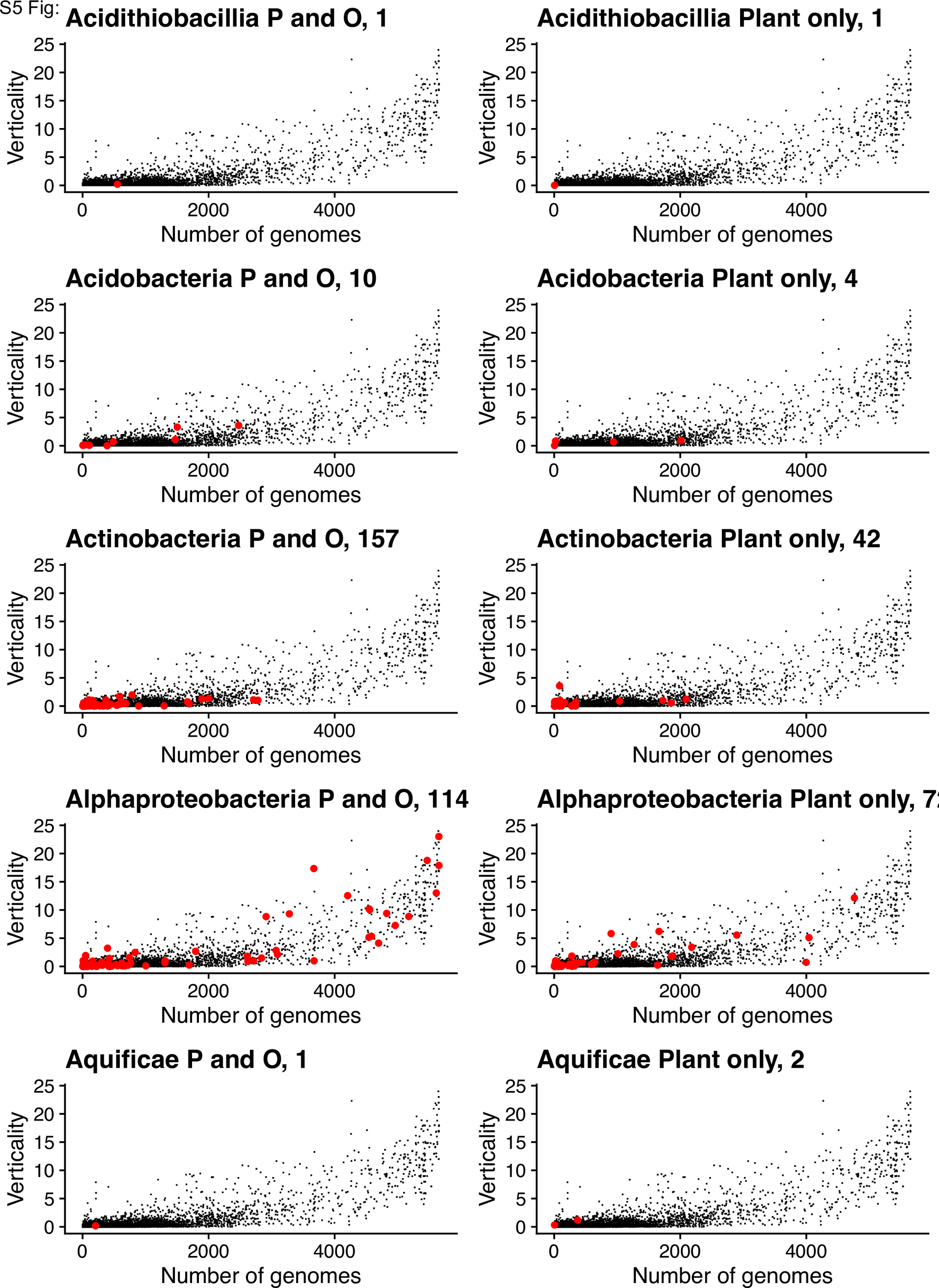

Supplement: S5 Fig — Mapping of EPCs to prokaryotic clusters. The EPCs were separated according to the pure sister group of eukaryotes in the trees and plotted in the same way as in Fig 4 of the main text. The left panel shows EPCs that may include all eukaryotic supergroups, the right panel shows only EPCs that include archaeplastidal eukaryotes. Meaning the latter are indicative of plastid endosymbiosis. For a better overview a headline is included in each plot that lists the taxonomic group represented, if it shows EPCs linked to the mitochondrial (‘P and O’, left panel) or to the plastidal endosymbiosis event (‘Plant only’, right panel), and the number of EPCs that are shown as red dots. (GZ) [file pgen.1009200.s015.tar.gz › S5_Fig/S5.1_Fig.tif]

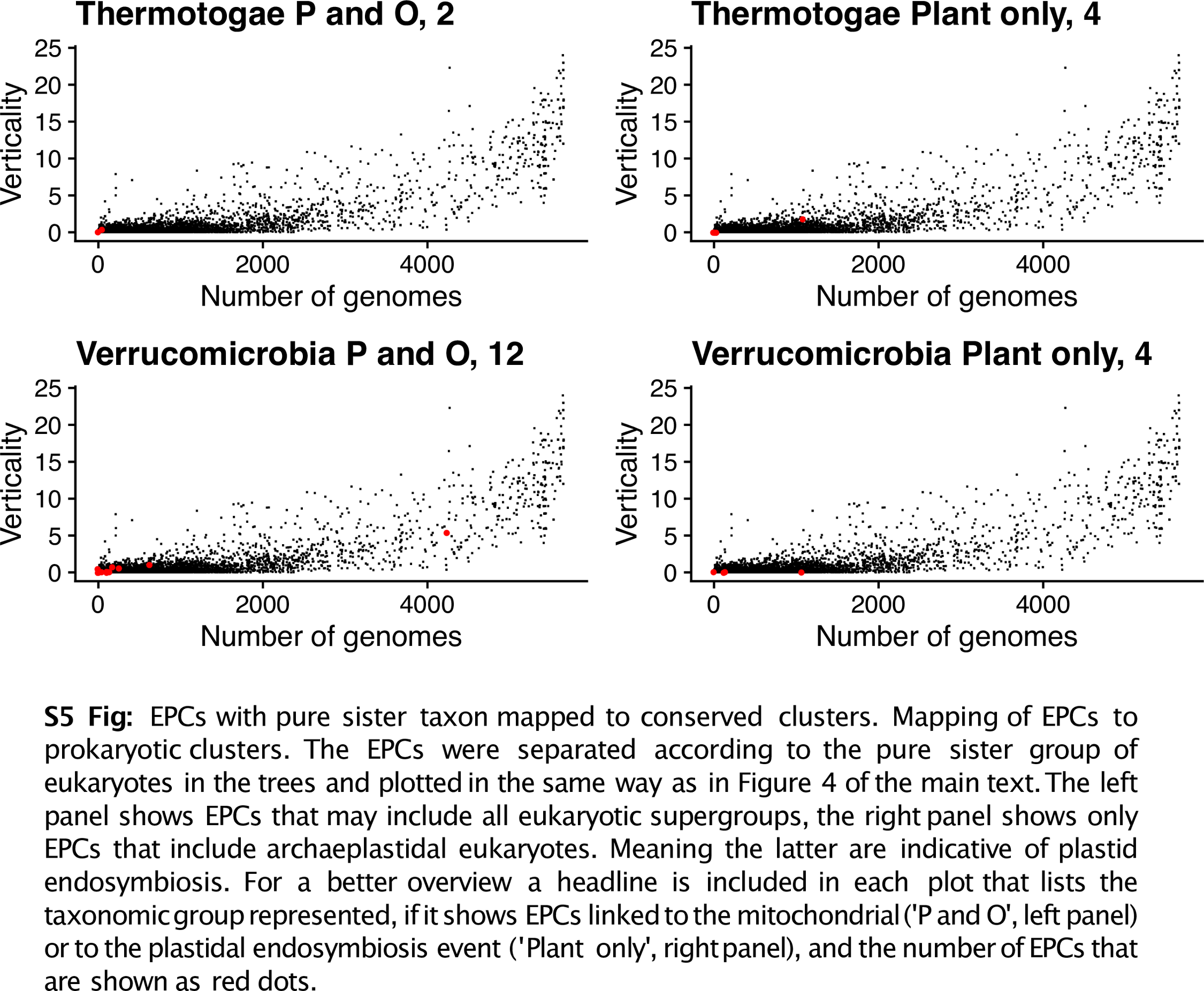

Supplement: S5 Fig — Mapping of EPCs to prokaryotic clusters. The EPCs were separated according to the pure sister group of eukaryotes in the trees and plotted in the same way as in Fig 4 of the main text. The left panel shows EPCs that may include all eukaryotic supergroups, the right panel shows only EPCs that include archaeplastidal eukaryotes. Meaning the latter are indicative of plastid endosymbiosis. For a better overview a headline is included in each plot that lists the taxonomic group represented, if it shows EPCs linked to the mitochondrial (‘P and O’, left panel) or to the plastidal endosymbiosis event (‘Plant only’, right panel), and the number of EPCs that are shown as red dots. (GZ) [file pgen.1009200.s015.tar.gz › S5_Fig/S5.9_Fig.tif]

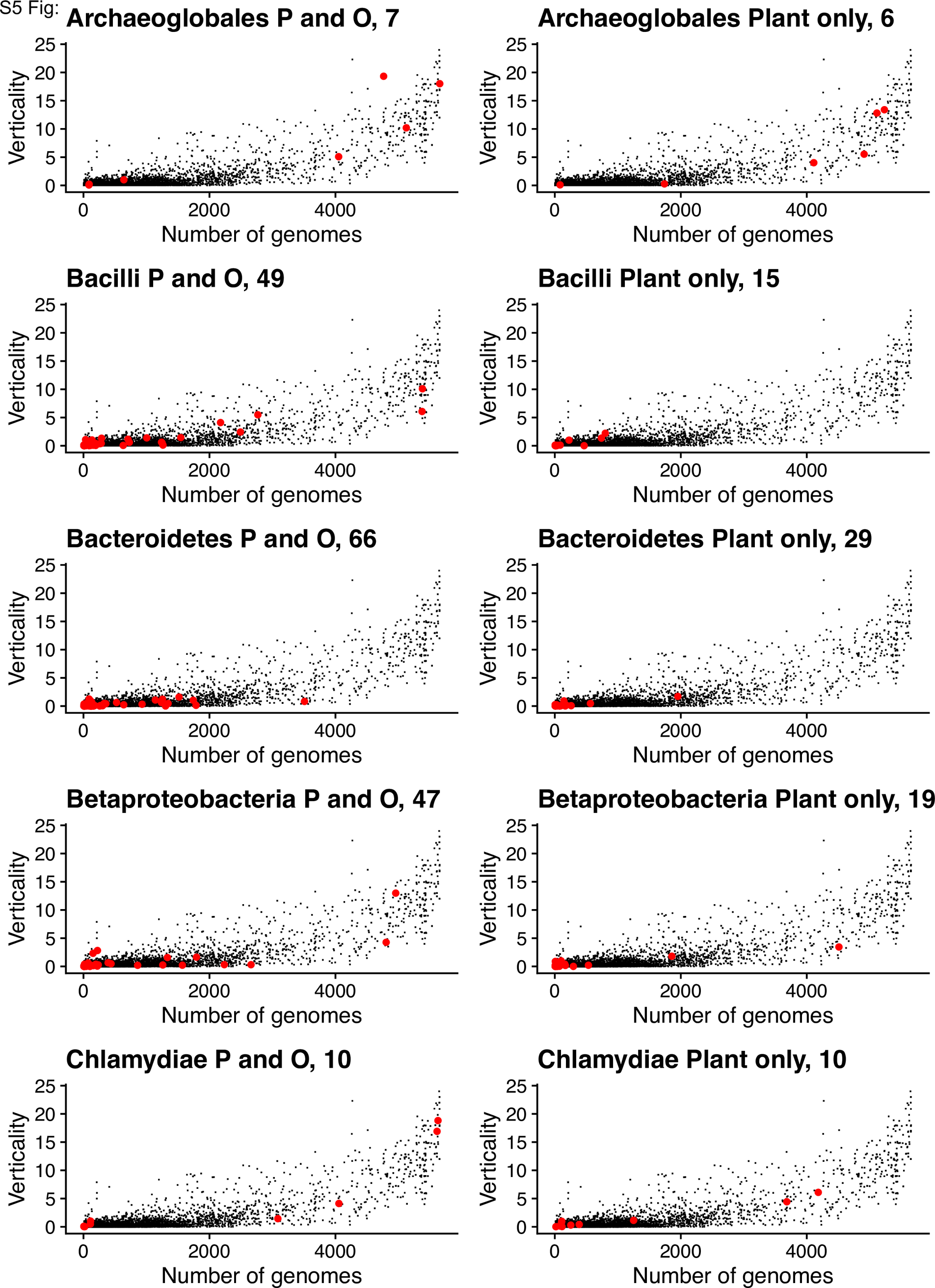

Supplement: S5 Fig — Mapping of EPCs to prokaryotic clusters. The EPCs were separated according to the pure sister group of eukaryotes in the trees and plotted in the same way as in Fig 4 of the main text. The left panel shows EPCs that may include all eukaryotic supergroups, the right panel shows only EPCs that include archaeplastidal eukaryotes. Meaning the latter are indicative of plastid endosymbiosis. For a better overview a headline is included in each plot that lists the taxonomic group represented, if it shows EPCs linked to the mitochondrial (‘P and O’, left panel) or to the plastidal endosymbiosis event (‘Plant only’, right panel), and the number of EPCs that are shown as red dots. (GZ) [file pgen.1009200.s015.tar.gz › S5_Fig/S5.2_Fig.tif]

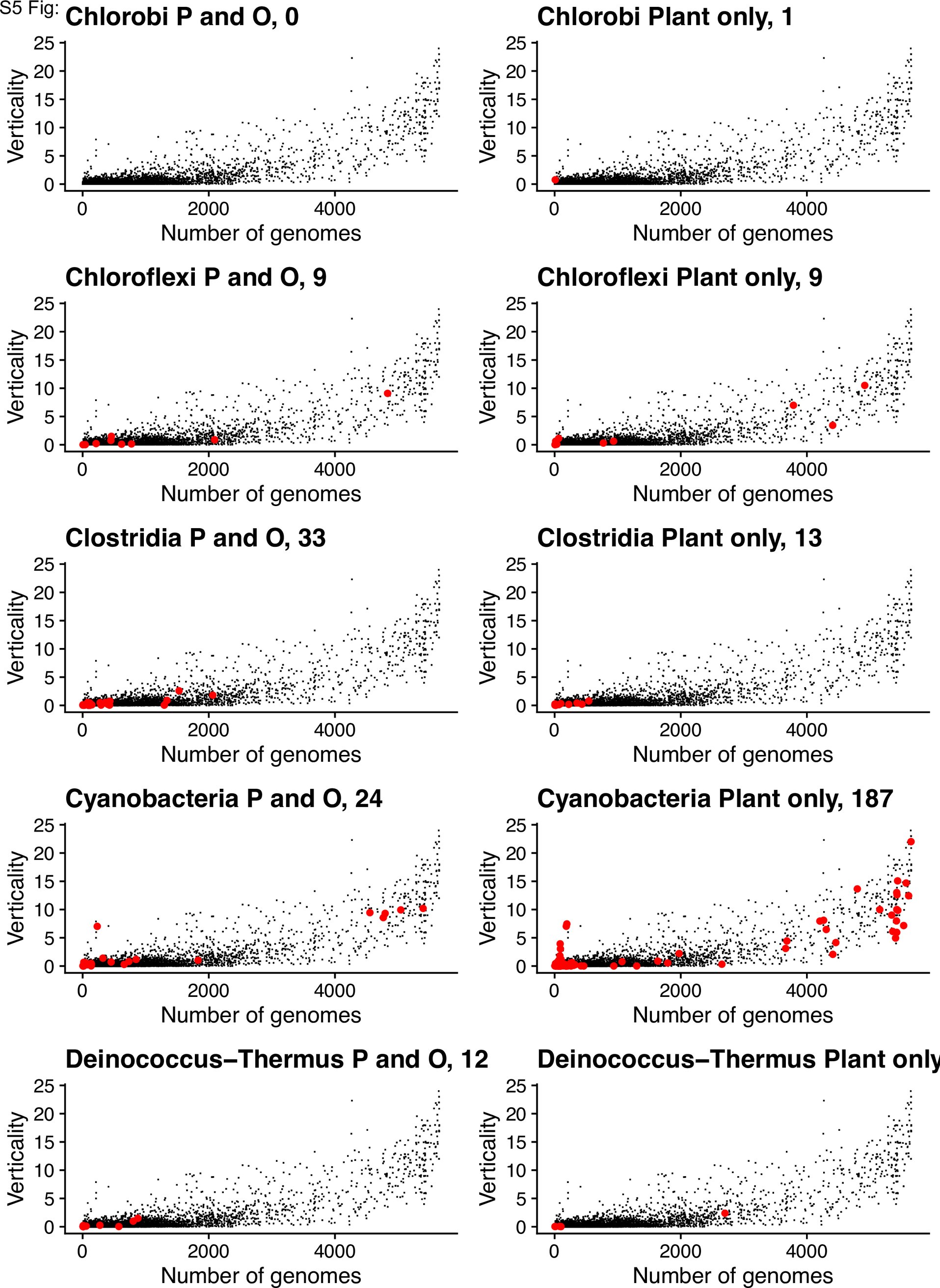

Supplement: S5 Fig — Mapping of EPCs to prokaryotic clusters. The EPCs were separated according to the pure sister group of eukaryotes in the trees and plotted in the same way as in Fig 4 of the main text. The left panel shows EPCs that may include all eukaryotic supergroups, the right panel shows only EPCs that include archaeplastidal eukaryotes. Meaning the latter are indicative of plastid endosymbiosis. For a better overview a headline is included in each plot that lists the taxonomic group represented, if it shows EPCs linked to the mitochondrial (‘P and O’, left panel) or to the plastidal endosymbiosis event (‘Plant only’, right panel), and the number of EPCs that are shown as red dots. (GZ) [file pgen.1009200.s015.tar.gz › S5_Fig/S5.3_Fig.tif]
