# Supplementary material for: A spectrum of verticality across genes
Source: PLoS Genet. 2020 Nov 2;16(11):e1009200. doi: 10.1371/journal.pgen.1009200 (PMC7660906; doi:10.1371/journal.pgen.1009200)
Supplement: S9 Table — (DOCX) [file pgen.1009200.s009.docx]

Supporting information:

| **Material** | **File name** | **Title** | **Appendix** | **Website** |
| --- | --- | --- | --- | --- |
| Table S9 | S9_Table.txt | List of all 101,422 RAxML-MAD rooted prokaryote-only trees employed in this analysis. |  | + |

Website:

http://dx.doi.org/10.25838/d5p-12
